# Research Progress on Small Molecular Inhibitors of the Type 3 Secretion System

**DOI:** 10.3390/molecules27238348

**Published:** 2022-11-30

**Authors:** Chao Lv, Ying Li, Yuxia Wei, Jiayu Wang, Hui Yu, Feng Gao, Chao Zhu, Xiangdi Jia, Mingqiong Tong, Pingxuan Dong, Qianqian Gao, Longlong Geng

**Affiliations:** 1 School of Chemistry and Chemical Engineering, Dezhou University, Dezhou 253023, China; 2 School of Life Sciences, Dezhou University, Dezhou 253023, China; 3 School of Medicine and Nursing, Dezhou University, Dezhou 253023, China

**Keywords:** virulence blocker, type 3 secretion system, T3SS inhibitors, biological activity assay, natural products

## Abstract

The overuse of antibiotics has led to severe bacterial drug resistance. Blocking pathogen virulence devices is a highly effective approach to combating bacterial resistance worldwide. Type three secretion systems (T3SSs) are significant virulence factors in Gram-negative pathogens. Inhibition of these systems can effectively weaken infection whilst having no significant effect on bacterial growth. Therefore, T3SS inhibitors may be a powerful weapon against resistance in Gram-negative bacteria, and there has been increasing interest in the research and development of T3SS inhibitors. This review outlines several reported small-molecule inhibitors of the T3SS, covering those of synthetic and natural origin, including their sources, structures, and mechanisms of action.

## 1. Introduction

Bacterial infections are one of the most common causes of human and animal disease [1,2]. Furthermore, infection by pathogenic plant bacteria can cause significant production losses in food and cash crops [3,4,5,6,7]. Generally, depending on the composition of the cytoderm, pathogens are classified into Gram-negative and Gram-positive bacteria. Various antibiotics and chemical antimicrobials have been developed for combating pathogenic bacteria [8]. However, due to their overuse, antimicrobial resistance (AMR) in pathogenic bacteria has become a concern. The World Health Organization has identified a list of pathogens that need particular attention regarding the development of new antimicrobial agents, especially those displaying AMR and multidrug resistance (MDR) [9]. These pathogens include the ESKAPE bacteria: *Enterococcus faecium*, *Staphylococcus aureus*, *Klebsiella pneumoniae*, *Acinetobacter baumannii*, *Pseudomonas aeruginosa*, and *Enterobacter* spp. (*Escherichia coli*), as well as the additional Gram-negatives *Salmonella enterica* and *Shigella flexneri* [9]. The need for novel antimicrobials is more pressing for Gram-negative bacteria, with only one drug having been successfully developed for Gram-negative infections in the past few decades [10]. Most new therapeutic agents against MDR Gram-negatives are in clinical and preclinical phases; therefore, research and development of novel drugs against MRD Gram-negative pathogens is urgently required [11,12].

## 2. Virulence Blockers

In the past decade, pharmaceutical chemists have focused on developing bactericidal or bacteriostatic drugs. With the increasing use of traditional antibiotics and chemical bactericides, pathogenic bacteria are under great pressure to survive. Selective pressure causes their mutation rates to accelerate, leading to serious drug resistance. However, to avoid the increasingly serious problem of bacterial resistance, scientists have studied drugs that do not directly kill bacteria. One of the important approaches for this is the development of virulence factor inhibitors [13,14,15]. The discovery of bacterial secretion systems was a milestone in bacterial pathogenic research [16]. Unlike antibiotics, which are already overused, virulence blockers inhibit pathogens by blocking their normal courses of infection. Although traditional antibiotics are effective against pathogens, they indiscriminately kill both pathogens and other members of the microbiota. However, virulence blockers do not inhibit bacterial growth; therefore, bacteria do not develop resistance due to selective pressures to survive. Virulence blockers have great potential as new anti-infective agents [17]. To date, several classes of virulence blockers have been developed and are even used in clinical practice [18,19,20].

The most commonly used virulence blockers are classified as antitoxins and are used against toxins secreted by pathogens, such as *Clostridium tetani*, *Corynebacterium diphtheriae,* and *Bacillus anthracis* [18,21,22,23]. These virulence blockers are usually antibodies, unlike most inhibitors in development. Such virulence blockers have been well researched and have been in use since the late 19th century [24,25]. Recently, specific molecules have been developed that inhibit the formation of cholera toxins and biofilms in *Vibrio cholerae* [26,27]. However, making bacteria “blind” to their neighbors may be a simpler strategy. Another potential method to reduce virulence involves targeting bacterial appendages and specialized secretion systems, which are requisite targets for scientists because they are necessary for growth in human hosts but not in other environments.

Virulence is the ability of pathogenic bacteria to cause diseases and is mainly related to virulence factors found in pathogenic bacteria. Common virulence factors include toxic molecules, such as toxins, cytolysins, and proteases, as well as toxic devices, such as the secretion systems, pili, and flagella [18]. To date, nine secretory systems have been identified and studied [28,29,30]. Type 3 secretory systems (T3SSs) are widely used by a variety of Gram-negative bacteria to infect host cells [31,32,33]; hence, they are one of the most studied virulence factors in pathogens. Most pathogens have multiple secretory systems as virulence factors. However, T3SSs are often the main virulence factor, including in animal pathogens, such as *E. coli*, *Chlamydia trachomatis,* and *Yersinia pestis*, as well as plant pathogens, such as *Xanthomonas oryzae, Dickeya dadantii,* and *Erwinia amylovora* [16,34,35].

## 3. Type 3 Secretion Systems

### 3.1. Components of T3SSs

Although T3SSs exists in a variety of pathogenic Gram-negative bacteria, they are given different names in different genera. For example, in pathogenic *Yersinia* bacteria, the T3SS is called Ysc (Yop secretion), and the functional Ysc secretion apparatus comprises approximately 20 *ysc* genes [36]. Yops (*Yersinia* outer proteins) are secreted by *Yersinia* T3SS [37] containing translocators (YopB, YopD, and LcrV) and effectors (YopH, YopE, YopT, YopO, YpkA, YopJ, and YopM) [38]. Here, we provide a general overview of the structural characteristics of T3SSs and discuss the specific inhibitory effects of the inhibitors reported.

T3SSs are protein complexes composed of multiple proteins that span the bacterial and host cell membranes, thereby establishing channels through which effectors are secreted immediately from the bacterial cytoplasm into the host cells [12]. T3SSs comprise over 20 proteins, usually evolved from flagella, and are similar in structure due to their highly conserved nature. T3SSs are encoded by 30–40 Kbp gene sequences that exist as pathogenic islands in bacterial chromosomes or plasmids [39]. The structures of various pathogen T3SSs are likened to syringes under transmission electron microscopy and thus are sometimes known as needle complexes (NCs) [40]. The needle structure, with the standard name of the T3SS injectisome, can directly inject the effector protein of the bacteria into the host cell.

**Figure 1:**
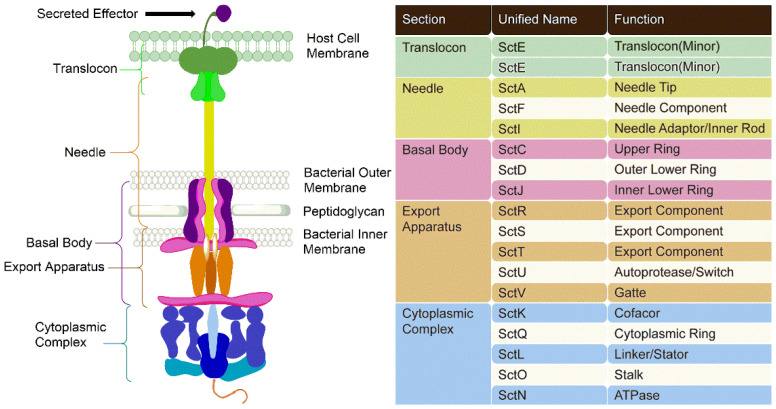
The general structure of the T3SS. (**Left**): Cross section of a prototypical T3SS with color-coded and labeled sections. (**Right**): Table of unified names of T3SS structural components by section [12,41].

Generally, the T3SS injection body can be divided into five main parts: translocon, needle, basal body, export apparatus, and cytoplasmic complex (Figure 1) [12,41,42].

The translocon (Figure 1, dark green) shows a protein hexamer hole that binds to the tip of the needle and inserts into the host cell membrane, through which secreted effectors enter the host cell [43,44,45,46]. Some consider the tip protein to be a part of the translocon, but in this paper it is considered part of the needle. Needles are tubular structures formed by the aggregation of helical monomers, and needle lengths vary as bacteria evolve to infect different host cells [47,48]. Furthermore, the length of the needle tip varies among different pathogen species. The T3SS of *E. coli*, for example, is special because the needle itself is shorter than the tip [49].

The T3SS attaches the needle to the bacterial cell surface via a basal body, a two-ring system comprising an inner ring (Figure 1, light purple) spanning the bacterial inner membrane (IM) and an outer ring (Figure 1, dark purple) that spans the bacterial peptidoglycan layer (PG) and the outer membrane (OM) [50]. The sorting platform comprises an export apparatus and a cytoplasmic complex (Figure 1, blue) that work together to sort, guide, and promote secretion [51]. The cytoplasmic complex has an ATPase (Figure 1, dark blue) that promotes the secretion of linearly unrolled proteins because folded proteins are too wide to pass through the ~2.5 nm needle [52,53]; therefore, the sorting platform can also serve as an effector recognition domain [54]. When secreted effectors enter the host cell, they cause a specific reaction, reprogramming the genetic sequence of the host so that bacteria can colonize it. Besides reprogramming (*Salmonella* spp.) [55], some effectors kill target cells (*Yersinia* spp.) [47]. There are over 30 unique effector genes in a particular pathogen [56,57,58].

### 3.2. Mechanism of Action of T3SSs

Although T3SSs have complex structures, they adopt a simple one-step and sec-independent mode of action [59]. The secretory signal is derived from the N-terminal 15–20 amino acid domain of the secretory protein and is independent of the signal peptide. When the pathogen comes into contact with host cells, the T3SS injection device is temporarily formed. Under physiological or low calcium levels, the device is activated and assembled and transports effector proteins into host cells, thus interfering with the normal function of host cells [60]. Various effector proteins from various pathogens have been confirmed to be latent substrates for T3SSs. The pathogenic effector protein T3SS can evade host defenses by altering signal transduction pathways. Some pathogenic effectors invade host cells and damage their immune system, resulting in host cell death [61]. Although the specific molecular mechanism of T3SSs is unclear, current studies suggest that the translocation of the T3SSs’ effector molecules is a pivotal pathway for Gram-negative pathogens to invade host cells [61].

**Figure 2:**
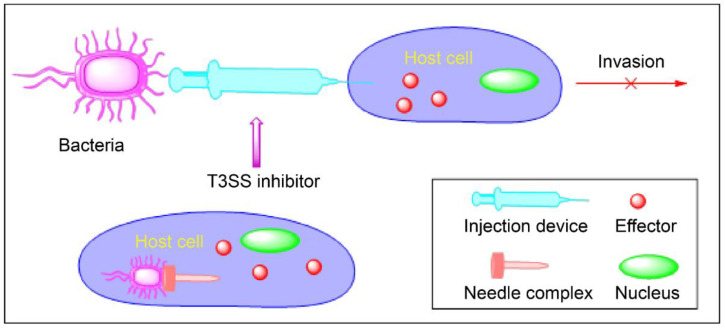
Schematic diagram of the anti-virulence strategy which uses T3SS inhibitors in Gram-negative bacterial pathogens.

T3SSs have become an important target for the development of novel drugs against Gram-negative bacteria. T3SS inhibitors inhibit the activity of T3SSs, blocking effectors from entering host cells through autonomous or passive immune pathways and preventing pathogens from infecting host cells (Figure 2). This mechanism of action of T3SS inhibitors is different from conventional antibiotics because they only target the virulence rather than the viability of bacteria, thus reducing the selection pressure of bacteria and the possibility of drug resistance. Therefore, the development of small-molecule T3SS inhibitors has received much attention recently [62,63,64]. In this paper, we will summarize the T3SS small-molecule inhibitors documented in the literature over the past two decades (2003 to 2022).

## 4. Inhibitors of T3SSs

Macromolecule inhibitors of T3SSs have been developed [65,66,67,68,69,70,71], including vaccination strategies, the development of anti-T3SS antibodies, and gene silencing approaches [72]. The most advanced anti-PcrV formulation (KB001) has entered phase IIa clinical trials [73,74,75]; meanwhile, the PcrV/Psl bispecific human monoclonal antibody MEDI3902 is currently in phase IIb clinical trials [76,77]. 

Small-molecule T3SS inhibitors are broadly divided into two types: synthesized inhibitors and naturally occurring inhibitors [78] and their derivatives. However, this does not rule out the presence of macromolecular inhibitors [79]. The T3SS inhibitor development process may involve high-throughput screening [80,81], computer-aided drug design [82], structural modification of lead compounds [83], and structure–activity relationship screening [81,84]. In this article, if the inhibitor molecule has a number in the literature, that number has been used to refer to it. If it has not been numbered previously, the numbering follows the order in which it appears in the article.

## 5. Synthesized Inhibitors of T3SSs

### 5.1. Salicylidene Acyl Hydrazides

**Figure 3:**
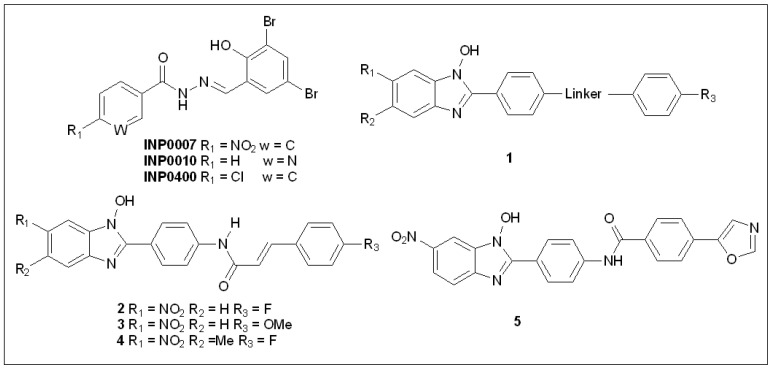
Structures of INP007, INP0010, INP 0400, and Compounds **1**–**5**.

In 2003, Kauppi et al. [85] discovered one salicylidene acyl hydrazide (Figure 3) which can intercept one or more effector proteins of T3SSs. Therefore, it was considered a first-class inhibitor of T3SSs. Kauppi et al. [85] screened a compound library with 9400 molecules and identified the salicylidene acyl hydrazide derivative INP007 as a T3SS inhibitor [86]. During screening, they used a luciferase reporter gene assay in viable *Y. pseudotuberculosis*, which revealed that the derivative inhibits T3SSs by lowering YopE translation levels and reducing luminescence. However, they found that these inhibitors do not notably inhibit bacterial growth in vitro but suggested that they act on T3SSs to some extent [16,85]. In their experiments, the salicylidene acyl hydrazide derivative INP007 and three other inhibitors with different structural skeletons were screened and the results of Western blotting demonstrated that an observed inhibition of actual Yop secretion corresponds to an inhibition of Yop expression.

To further study the inhibitory activity of these compounds on T3SSs, Elofsson et al. prepared and characterized several analogs of INP007 [87]. This study suggested that INP007 specifically targets the *Yersinia* T3SS and blocks the translocation of Yop effectors, leading to chemical decay similar to that of secretory and translocation deficient mutants. To some extent, the effect of these derivatives was similar to that of calcium on Yop secretion. Furthermore, HeLa cells infected with bacteria were used as models and INP007 inhibited the translocation of the Yop effector but had no inhibitory effect on either HeLa cell or bacterial growth [87].

These compounds can inhibit the T3SS of *Yersinia* and other pathogens. Keyser et al. [14] found that compound INP0010 blocked Yops secretion into the cytoplasm of target cells and restrained the duplication of *Chlamydia* in HEP-2 cells. Another derivative INP0400 also inhibited the T3SS of *Chlamydia*, but by a different mechanism. Muschiol et al. [88] found that intracellular duplication and infection of *C. trachomatis* is inhibited by the compound INP0400 at micromolar concentrations, resulting in small inclusion bodies typically involving one or several reticular bodies (RBs). In the event of infection, high concentrations of INP0400 partially block the entry of elementary bodies into host cells. Since early treatment of mammals inhibits the localization of the mammalian protein 14-3-3β in inclusion bodies, this suggests that the inclusion membrane lacks the early inducible T3SS effector IncG. Experiments with INP0400 on midlife *Chlamydia* showed that the compound blocked the secretion of the TTS effector IncA and homogeneous vesicle fusion mediated by the protein. NP0400 can lead to the separation of RBs from the inclusion membrane and inhibit the transformation of RB into elementary body conversion, resulting in a significant decrease in infectivity, which can be observed in experiments using NP0400 to treat *Chlamydia* in the late life cycle [88]. Salicylidene acyl hydrazide has also been studied for its inhibitory effect on T3SSs of other Gram-negative bacteria [79,89,90]. Hudson et al. [90] treated *Salmonella* with two salicylidene acyl hydrazide derivatives and found that they had an inhibitory effect on the T3SS of *Salmonella* by inhibiting the secretion of effectors and blocking the erosion of cultured epithelial cells, suggesting that these two compounds may prevent enteritis in vivo. This indicates these compounds can interdict the T3SS in several pathogens, so they have the potential to be used as novel drugs against Gram-negative bacteria [14].

### 5.2. N-Hydroxybenzimidazoles

In *Y. pestis* and *Y. pseudotuberculosis* cells, virulence is regulated by LcrF, which is a transcription factor of the multiple adaptational response (MAR) [91]. LcrF is expressed when the bacterium encounters host cells or when the temperature changes, and this expression activates the expression of *Yersinia* cytotoxic effectors Yops, including YopH, YopJ, and YopE [36,92]. Kim et al. [91] synthesized a series of hydroxybenzimidazoles (molecular skeleton **1** in Figure 3) and found that some of these compounds could inhibit the T3SS of *P. tuberculosis* by inhibiting LcrF, and the structure–activity relationship (SAR) was further examined. Some molecules they selected showed inhibitory effects when aggregated in an initial cell-free LcrF-DNA binding assay and later in unbroken cells. Since these compounds do not kill bacteria extracorporeally, it is suggested that they have no adverse effect on bacterial growth. The experimental results show that *N*-hydroxybenzimidazole compounds **2**, **3**, and **4** can effectively inhibit the virulence of bacteria and have the potential to prevent bacterial infection caused by *Yersinia* spp. [91]. The authors performed ExsA-DNA and SlyA-DNA binding experiments on these compounds (**2**, **3**, and **4** with IC_50_ ≤ 10 μM) to verify the specificity of the target. It was found that ExsA was highly homologous with LcrF in the DNA-binding domain (85% homogeneity, 92% similarity). Theoretically, if these molecules target the DNA-binding domain of the LcrF protein, then their binding to Exsa-DNA would have similar binding effects. The inhibitory activities of these three compounds against LcrF and ExsA are similar, as reflected by the IC_50_ values. The authors used the SlyA-DNA binding assay to test the specificity of the LcrF inhibitors because it belongs to the MarR family of transcription factors in *Salmonella* spp., which differs from that of the MAR proteins. As expected, in the SlyA-DNA binding assay, no LcrF inhibitors displayed binding activity. These observations demonstrated the target specificity of *N*-hydroxybenzimidazole inhibitors for MAR transcription factors. Compounds **2**, **4**, and **5** showed no significant inhibitory activity against *P. tuberculosis*, *S. aureus,* or *E. coli* in in vitro antibacterial tests, confirming that they had little effect on bacterial growth. In in vitro experiments, a series of *N*-hydroxybenzimidazole inhibitors exhibited an inhibitory effect against the cytotoxicity of T3SS-dependent *Y. pseudotuberculosis* against macrophages.

In mice infected with *Y. pseudotuberculosis* pneumonia, when treated with molecules **2** and **5,** these two molecules reduced the bacterial load in the lungs and provided a significant survival advantage [80]. As transcription factors of the MAR family are conserved, its members play a central role in the pathogenesis of the whole bacterial genus. Therefore, these inhibitors have wide applicability [93].

### 5.3. Phenoxyacetamides

**Figure 4:**
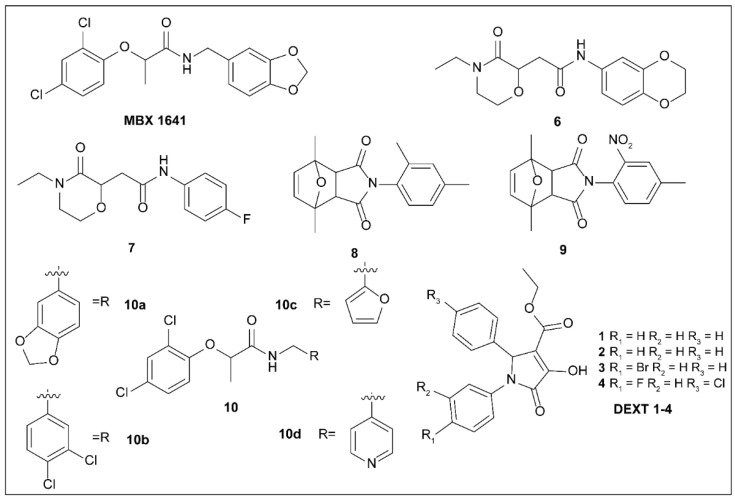
Structures of phenoxyacetamides, **10a**–**d**, and DEXT 1–4.

T3SSs are also implicated in disease severity associated with *P. aeruginosa* infections, especially in patients with compromised immune systems. Studies have identified the biochemical and enzymatic functions of the effectors ExoU, ExoS, and ExoT of T3SSs, but the relative roles of these proteins in the pathogenesis of acute infection remain unclear. As ExoU and ExoS are usually produced by different strains, it is difficult to directly compare the roles of the two proteins in infection. Hauser et al. [94] created mutant strains of ExoU, ExoS, and ExoT to evaluate the mechanism of action of each effector protein during the pathogenesis of acute pneumonia in mouse models. The results showed that ExoU secretion had the greatest effect on virulence, ExoS had a medium effect, and ExoT had a minor effect. These are the first confirmations that ExoS is a major virulence factor [95]. Moir et al. [64] applied two cellular reporter assays to screen the activities of a library of 80,000 molecules. The initial experiment began with the reliance on transcription of T3SS operons on the T3SS-mediated secretion of a negative regulator and comprised transcriptional fusion of the *Photorhabdus luminescens luxCDABE* operon to the *P. aeruginosa exoT* effector gene [96]. Further tests were performed to detect natural ExoS secretion and T3SS-mediated secretion of *P. aeruginosa* ExoS-β-lactamase fusion protein [96]. Activity screening revealed five compounds (MBX1641 and compounds **6**–**9**) (Figure 4) with minimal cytotoxicity that could selectively inhibit T3SS and did not affect the normal growth of pathogenic bacteria. Further studies showed that T3SS-mediated secretion of *Y. pestis* YopE-β-lactamase fusion protein was blocked by the action of these inhibitors [96]. Furthermore, phenoxyethamide MBX1641 also blocks the T3SS-mediated entry of effectors into cultured mammalian cells and is considered the most promising inhibitor. A preliminary SAR study of the phenoxyethamide series showed that the R configuration of the chiral center is important for activity; however, various substituents on one of the two aromatic rings are tolerated and do not result in loss of potency [96].

To obtain more effective T3SS inhibitors of *P. aeruginosa*, Chen et al. collaborated with Yang et al. to conduct an in-depth SAR study on MBX1641 [97]. They synthesized a group of new α-phenoxyethamide analogs based on the MBX1641 structure by modifying the amide substituent by introducing different R groups and changing the length of the alkyl chain linked to the R group [97]. After activity testing, four new derivatives (compounds **10a**–**10b**), shown in Figure 4, showed inhibitory activity against the exoS gene expression of *P. aeruginosa*. Compared to MBX1641, compound **10d** not only showed stronger inhibition of the T3SS of PAO1 but also had better aqueous solubility [97].

Furthermore, Western blotting demonstrated that the expression of ExoS and ExoT effectors was significantly blocked by MBX1641, **10a**, and **10d**. Laboratory findings have indicated that these compounds can potently inhibit the expression of effectors of PAO1 T3SS ExoS and ExoT.

Recently, Aburto–Rodríguez et al. [98] prepared four pyrrolidones DEXT 1–4 (Figure 4). The secretion of effector proteins ExoU and ExoT, cluster movement, and biofilm formation of pathogens were inhibited when treated with these four compounds. Treatment with DEXT-3 alone or in combination with furanone C-30 (a quorum sensing inhibitor) or MBX-1641 on *P. aeruginosa* PA14-induced necrosis models led to reduced necrosis [98].

### 5.4. 2-Imino-5-Arylidenethiazolidinones

**Figure 5:**
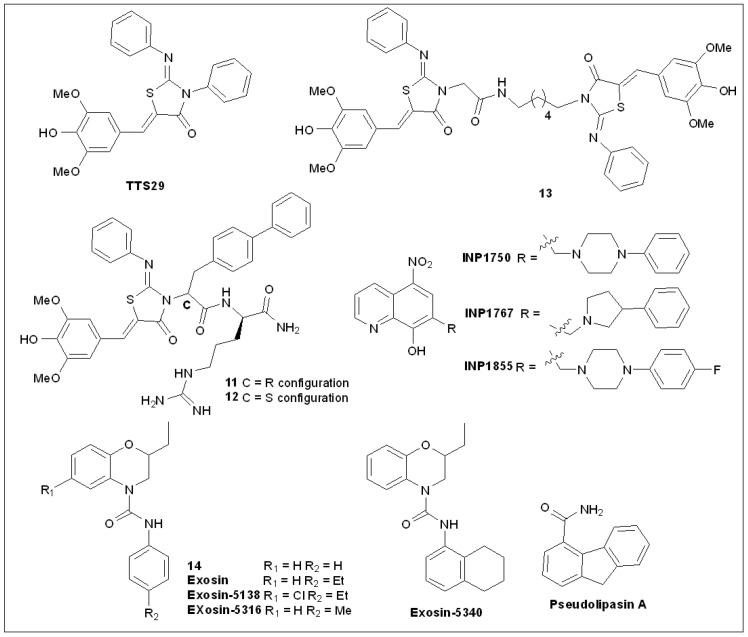
Structures of TTS29, **11**, **12**, **13**, IPN1750, IPN1767, IPN1855, exosin and analogs, and Pseudolipasin A.

To find new T3SS inhibitors to block *Salmonella* infection, Felise et al. [99] conducted high-throughput screening of a small-molecule library and found a new class of T3SS inhibitors with 2-imino-5-arylidene thiazolidinone scaffolds (Figure 5). These molecules showed T3SS inhibitory activity that inhibits the secretion of virulence factors specifically in Gram-negative bacteria that cause disease in plants and animals. The compounds showed inhibition of the T3S-dependent function (except flagellar movement) and T2S-dependent function, implying that its binding site may be the OM domain conserved between the two secretory systems [96]. The results showed that the compound exhibited extensive inhibition of the secretion systems of various Gram-negative bacteria, for example, *Y.* spp., *P. aeruginosa,* and *Francisella Novicida*, and can therefore prevent and treat a variety of bacteria-induced diseases. However, inhibition of T3SS by TTS29 was only shown at an IC_50_ of 83 μM, which limited its further development.

To develop more effective inhibitors, Kline et al. [100] synthesized a series of novel derivatives of TTS29 using a solid-phase method. SAR studies showed that the substitution patterns on the imino nitrogen, aryl groups, and aryl rings were key to the inhibitory activity, whereas the amino nitrogen was tolerant to modification.

The IC_50_ values of the dipeptide analogs **11** and **12** against T3SS were 8 μM and 3 μM, respectively, and the inhibitory effect was significantly better than that of TTS29 [100]. In a subsequent study, they reported a series of thiazolidone analogs in which the heterocyclic rings are linked by long alkyl chains as dimers. Many of these dimers inhibit the secretion of T3SS-dependent virulence proteins at concentrations lower than those reported for the original monomer molecule TTS29. For example, dimer **13** inhibited T3SS with an IC_50_ value of 5 μM [101].

### 5.5. 8-Hydroxyquinoline Derivatives

Enquist et al. [102] screened a library of 17,500 small molecules for inhibition of T3SSs and surprisingly found only one hit, the 8-hydroxyquinoline derivative INP1750. A small-molecule inhibitor is itself insufficient for activity exploration; hence, a series of analogs of INP1750 were synthesized using classic Mannich chemistry [102]. INP1750, INP1767, and INP1855 were the three most active of these molecules (Figure 5). They were then used to study the effects on bacterial growth and the inhibition of phosphatase activity of the secreted YopH described above [102]. It was revealed that these molecules displayed dose-dependent effects on the inhibition of the reporter gene and YopH, with EC_50_ values ranging from 6 to 12 μM. Subsequently, the three most promising compounds in the macrophage infection model were further evaluated.

Wild-type *P. tuberculosis* uses T3SSs to transfer toxins into the cytoplasm of macrophage J774, ultimately leading to cell death or reduced viability. In healthy cells, calcitonin AM is converted into a green fluorescent molecule that can be used to monitor the viability of J774 cells. The bacteria cause a decrease in cell viability, which was attenuated by the addition of the three inhibitors. Uninfected macrophages treated with the above inhibitors showed moderate cytotoxicity, and they had almost no effect on macrophages infected with non-toxic T3S-defective mutants [102,103]. Previous studies have shown that T3SS inhibitors that work against *Y*. *pseudomonas* are active against a variety of bacteria, including *Chlamydia* [92]; therefore, these three compounds were evaluated against *C. trachomatis* using the in vitro infection model [103,104]. The minimum inhibitory concentrations (MICs) of the three compounds against *C. trachomatis* ranged from 3 to 25 μM, and resazurin was used to evaluate cell viability at double the MIC concentrations. INP1750 was found to have some cytotoxicity in *Yersinia* infection models, whereas the most effective compounds INP1767 and INP1855 were found to have cell survival rates of over 90%. The effect of INP1855 on the growth of *C. trachomatis* was further observed by fluorescence microscopy after immunostaining. The results showed that when the concentration of INP1855 was 1.56 μM, the intracellular inclusion size decreased slightly compared to the dimethylsulfoxide (DMSO) control, whereas when the MIC concentration was 3.13 μM, there was no intracellular inclusion [102].

### 5.6. 2,3-Dihydro-4H-benzo[b][1,4]oxazine-4-carboxamides

*P. aeruginosa* infection is the main cause of death in patients with cystic fibrosis, and the bacterium has shown remarkable levels of antibiotic resistance. Inhibition of ExoS, a toxin that plays a key role in *P. aeruginosa* infection of host cells, may help block infection by the bacterium. Arnoldo et al. [105] screened the first small-molecule inhibitor of ExoS (Figure 5) based on a novel approach that combined chemical and genetic strategies to interfere with yeast. One such inhibitor is Exosin, which may modulate the enzyme activity of the toxin. Further studies showed that Exosin could block *P. aeruginosa* from infecting mammalian cells. Together, these results demonstrate the feasibility of a yeast-based approach to the discovery of new antibiotics. These inhibitors can be used as lead compounds in the development of new antibacterial drugs, and similar development strategies can be used for other pathogens [105].

Considering the structural specificity of Exosin, the study screened 50 molecules in yeast similar in structure to compound **14** hoping to find inhibitors with higher potency against ExoS ADP-ribosyl transferase (ADPRT) activity. Seven analogs showed better potency than compound **14**. The flow cytometry results showed that these molecules had protective effects on CHO cells invaded by *P. aeruginosa*. However, only Exosin-5138 and Exosin-5316 showed protection by lactate dehydrogenase (LDH) detection. Therefore, only these two compounds were used for further activity studies.

As compound Exosin-5340 showed no protection in infected yeast and CHO cell tests, they were selected as negative controls. It was found that the substituent transformation on the phenyl group had a great effect on the inhibitory activity of the compound. Three analogs, Exosin-5138, Exosin-5316, and Exosin-5340, were chosen for quantitative and IC_50_ determination of yeast growth recovery. Exosin-5138 restored 36.8% of yeast growth, making it almost twice as protective as Exosin, which did not provide protection. Exosin-5316 restored yeast growth by 27.4%, and Exosin restored yeast growth by 20.5%, showing a similar protective effect [105]. The results of the fluorescence ADPRT enzyme assay showed that these three compounds directly regulated the ADPRT activity of ExoS. The IC_50_ values of these compounds were tested, and these values were consistent with the effects of the small molecules tested in the yeast model, among which Exosin-5138 and Exosin-5316 had the strongest effects, while Exosin-5340 had the weakest effect. The activities of these analogs were then studied using mammalian cell models. The protective effects of Exosin-5138 and Exosin-5316 on CHO cells were tested. The dot plot of Exosin-5138 showed that the number of dead cells decreased significantly when the compound concentration was 80 μM, indicating that exosin-5138 had a greater inhibitory effect on ExoS cytotoxicity than the original. Furthermore, flow cytometry showed that Exosin-5316 had a protective effect on cells.

CHO cell death/near-death was significantly reduced by the addition of the analog Exosin-5316, which exhibited similar protection to the initial compound. These data showed that Exosin and its analogs had similar effects in yeast and infected CHO cells, making yeast an effective detection system. The system can assess the effectiveness of the original inhibitors, ranking the activities of the inhibitors before subsequent trials in more complex infection models [105].

Exosin inhibits the enzyme activity of ExoS ADPRT by acting as a competitive inhibitor of NAD^+^ substrates, reducing the virulence of pathogenic bacteria to mammalian cells and not affecting bacterial strains expressing other effector factors [105,106].

### 5.7. Pseudolipasin A

Effector proteins are delivered to the host cytoplasm by multiple pathogens via the T3SS pathway. Exoenzyme U (ExoU) is a potent cytotoxin expressed by some strains of *P. aeruginosa* and directly enters the host cytoplasm through the T3SS pathway. After entering mammalian cells, invading cells are rapidly lysed by the phospholipase A2 (PLA2) activity of ExoU.

To screen for ExoU inhibitors in mammalian cells, Lee et al. [107] developed a system that took all potential reductions in *P. aeruginosa*-infected cells as indicators of cell viability. After screening a library of 50,000 compounds, they found a series of compounds that could protect CHO cells from cleavage by ExoU-expressing *P. aeruginosa*. The most effective compound was *Pseudomonas* phospholipase inhibitor A (Pseudolipasin A) (Figure 5) [107]. Pseudolipasin A may be used to explore the mechanism of intracellular PLA2 activity in ExoU. One possible mechanism of Pseudolipasin A is interdiction of the PLA2 activity of ExoU; therefore, it has no discernible effect on eukaryotic cell PLA2. To evaluate whether Pseudolipasin A-mediated killing was exclusive to CHO cells, they examined other T3SSs killing sensitive eukaryotic cell models. Co-culture of *D. discoideum* and *P. Aeruginosa* PA103 on agar with Pseudolipasin A can make *Discococcus* immune to *P. aeruginosa* PA103 and form visible plaques. Thus, Pseudolipasin A has a protective effect on *D. discoideum* with an IC_50_ value of 0.2 μM. The addition of methyl arachidenyl fluorophosphate (MAFP) [108], an analog inhibitor of cytoplasmic PLA2, to the medium also inhibited ExoU PLA2 activity, but did not protect *D. discoideum* from PA103. Therefore, MAFP, a known PLA2 inhibitor, does not protect eukaryotic cells from ExoU-dependent killing, but Pseudolipasin A does, suggesting that Pseudolipasin A is more specific to ExoU. These tests also indicated that Pseudolipasin A has a protective effect on many eukaryotic cells. The screening of ExoU PLA2 inhibitors provides directions for research in three areas, namely, elucidation of the newly observed function of PLA enzymes, confirmation of the activity of the ExoU enzyme, and the development of new therapeutic methods [108]. Recently, Foulkes et al. [84] developed a pipeline that combines ExoU inhibitors with certain antibiotics as a new method to treat infections caused by ExoU-producing *P. aeruginosa* and to identify more effective ExoU inhibitors.

### 5.8. Arylsulfonamides

**Figure 6:**
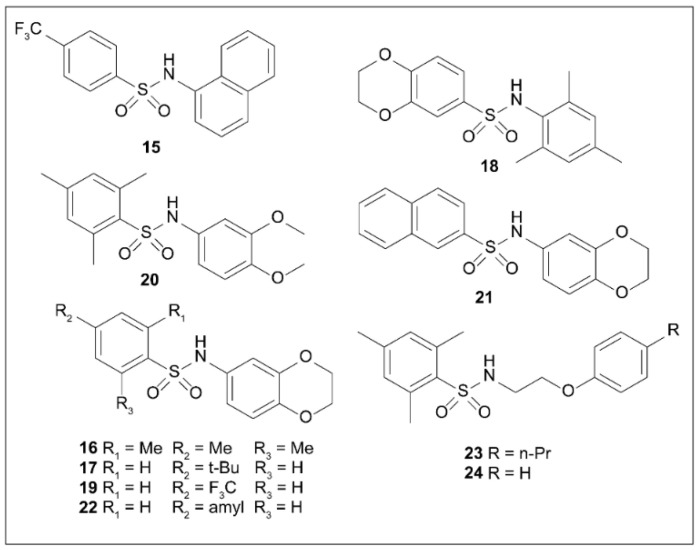
Structures of arylsulfonamides.

Kim et al. [109] screened an internal complex library using a yeast-based screening system and found that some sulfonamides (Figure 6) showed important ExoU-inhibiting activity, but these compounds showed non-specific cytotoxicity.

Several novel sulfonamides showed ExoU-inhibiting activity, and intermediate compound **15** was confirmed to block ExoU-dependent cytotoxicity. The non-specific cytotoxicity was measured before the activity was assessed, and none of the compounds tested for activity showed non-specific cytotoxicity to yeast or HEK293T cells. Of these synthetic sulfonamides, trimethylphenyl (**16**) and 4-tert-butylphenyl (**17**) derivatives were the most active. Compounds **15**, **19**, and **21** also showed moderate activity. Compared to compound **16**, the activity of reverse sulfonamide **19** was significantly decreased [109]. The 3,4-dimethoxy substituted compound **20** and amyl substituted compound **22** were found to be inactive. Phenoxyethyl substitution reduced the activity, with compounds **23** and **24** showing only slight activity. These results suggest that the benzodioxane moiety contributes to the activity. Most molecules with ExoU-dependent cell death exhibit cytotoxicity in HEK293T cells. Compound **24**, with weak activity, showed moderate cytotoxicity in HEK293T cells [109]. The discovery of such compounds contributes to the enrichment of ExoU inhibitors.

### 5.9. Epiphepropetin D Derivatives

The T3SS is rarely required by non-pathogenic Gram-negative bacteria, and there are dozens of Gram-negative bacteria that require the T3SS for toxicity, making it a promising target for toxicity blockers. Mammalian cells recognize functional T3SSs and activate NF-κB, which offers a quick and ingenious means for the detection of T3SS inhibitors.

**Figure 7:**
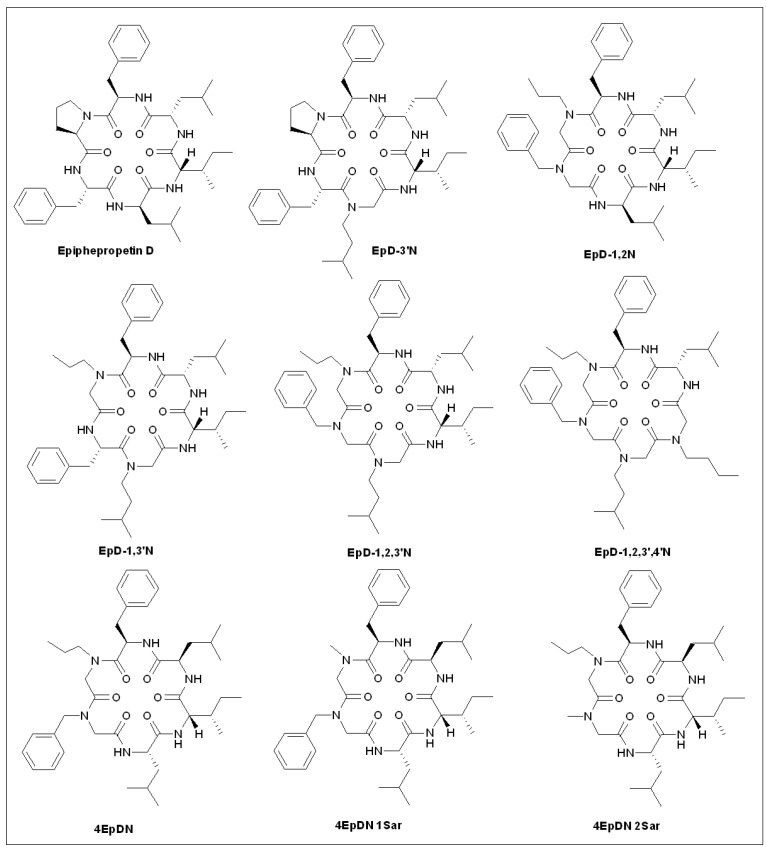
Structures of epiphepropeptin D derivatives.

Duncan et al. [110] tested the T3SS activity of *Yersinia* using a luciferase assay driven by NF-κB and successfully screened a T3SS-inhibitor family named Piericidins (Figure 7). To increase detection efficiency, they constructed a stable HEK293 cell line expressing the green fluorescent protein (GFP) reporter gene driven by NF-κ B. Using this cell line, they identified a group of eight small molecular cyclic peptides that inhibit T3SS secretion in *Yersinia* and *P. aeruginosa*. According to the structural transformation of natural product Phepropeptin D [111] (Figure 7), a compound screening library was synthesized containing 20 synthetic cyclic peptides and their activities were assessed. The compounds EpD-3′N, EpD-1,2N, EpD-1,3′N, EpD-1,2,3′N, and EpD-1,2,4′N were not toxic to mammalian cells and did not inhibit bacterial growth at 240 μM. However, over 40% inhibition of the translocation of Yersinia YopM effector protein into mammalian cells was observed at 7.5 μM. Compound EpD-1,2,4 ′N can inhibit the T3SS secretion of *Yersinia* and *Pseudomonas* and thus block the invasion of *Yersinia* T3SS effector protein into target host cells [112].

Recently, the same group synthesized a series of novel Phepropeptin D derivatives and used them to study the inhibition of T3SS [83]. Among them, the IC_50_ value of 4EpDN against T3SS (4 mM) was half that of the lead compound, Phepropeptin D. In addition, 4EpDN not only inhibited the T3SS of *Y. ysa* but also showed inhibitory activity against SP-1 T3SS of *Salmonella*, indicating that it had a broad spectrum of inhibition [83]. Furthermore, 4EpDN intensively inhibited the growth of *C. trachomatis* in HeLa cells, which required the participation of T3SS. The compound 4EpDN specifically blocks injection T3SS but not flagellar T3SS because it does not inhibit the secretion of *Salmonella* flagellar T3SS substrates [83]. Collectively, 4EpDN specifically inhibits the injection of T3SSs from several Gram-negative bacteria, providing a purposeful reference for the development of novel inhibitors of T3SSs.

### 5.10. Fluorothiazinon (FT)

**Figure 8:**
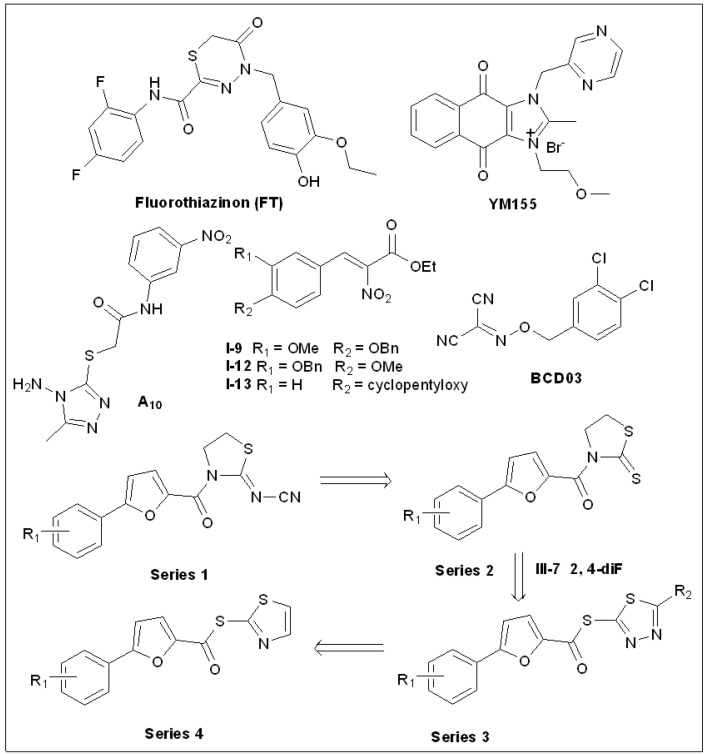
Structures of Fluorothiazinon (FT), YM155, A_10_, Thiazolidin derivatives and analogs, I-9, I-12, I-13, and BCD03.

To develop novel T3SS inhibitors against chronic *Chlamydia* infection, a series of novel compounds were synthesized by Zigangirova et al. [113], including fluorothiazinon (F, named as CL-55 in their study; Figure 8). CL-55 blocked the translocation of the T3SS effector protein IncA of *C. trachomatis* using specific antibodies. The number of particles infected with *C. trachomatis* decreased in a dose-dependent manner with CL-55, and the activity of particles infected with *C. trachomatis* was inhibited when the concentration reached 50 μM. Quantitative real-time PCR was used to evaluate the expression of the *lcr*E gene that encodes the T3SS regulatory protein and a 90% reduction in the presence of 50 µM of CL-55 was observed [113]. It was found that CL-55 has universal applicability to microorganisms of the Chlamydiaceae family, and 50 μM of CL-55 restrained the accumulation and survival capacity of *C. pneumoniae* and *C. muridarum* in McCoy cells. It is worth noting that FT is in phase II clinical trial; however, clinical trial results are not yet available [77]. Further laboratory work is necessary to understand how the inhibition works.

In 2016, Nesterenko et al. [114] studied the inhibitory effect of CL-55 on the *S. enterica* Serovar Typhimurium T3SS. The effects of CL-55 on bacterial growth were studied using the plate counting method. CL-55 was found to not significantly block the growth of the *S. enterica* Serovar Typhimurium IV147 strain when the concentration was up to 200 μM compared to the control. In vivo experiments showed that CL-55 promotes the survival rates of BALB/cJCit effectively. Acute and chronic toxicities confirmed that a single dose of 5000 mg kg^−1^ of CL-55 did not lead to mortality or toxic changes in the organs of mice and rats. In addition, CL-55 was found to have no carcinogenic, teratogenic, or mutagenic effects on experimental animals. Pharmacokinetic studies have shown that CL-55 is quickly circulated by the bloodstream into all organs [114]. Taken together, these results indicate that CL-55 can be used to effectively treat *S. typhi* infections and that it displays a good safety profile, which makes it a promising new anti-infection drug. Recently, Zigangirova et al. found that FT (CL-55) showed significant effects in the treatment and prevention of *Salmonella* infection and even eradicated Salmonella infection in mice [115]. FT(CL-55) has great potential as a clinical candidate for antimicrobial therapy.

### 5.11. Sepantronium Bromide (YM155)

Glycosyltransferases NleB and SseK are effector proteins of T3SS and can glycosylate arginine residues of protein substrates. Zhu et al. [116] conducted a high-throughput screening of a library of 42,498 molecules to find NleB/SseK inhibitors. The glycosylation assays found that YM155 (Figure 8) inhibits NleB/SseK in a variety of bacteria, including *E. coli* NleB1, *Citrobacter rodentium* NleB, and both *S. enterica* SseK1 and SseK2. Growth experiments have confirmed that 125 μM of YM155 has no significant effect on *C. rodentium*, EHEC, or *S. enterica*, and that, at certain concentrations, it can reduce *Salmonella* survival in RAW264.7 cells [116]. These experiments confirmed that YM155 may be a useful inhibitor of the virulence factor.

### 5.12. 1,2,4-Triazole Thioether(A_10_)

Inhibitors of plant pathogenic virulence factors can effectively alleviate plant microbial diseases. Shao et al. [117] synthesized a series of 1,2, 4-triazole and 1,3, 4-oxadiazole compounds (Figure 8) to screen for T3SS inhibitors of *Xanthomonas oryzae pv. oryzae* (Xoo). Activity screening showed that A_10_ could significantly down-regulate the expression of the T3SS and transcription activator-like effector correlative proteins of Xoo. Furthermore, A_10_ can initiate and inhibit the transcription levels of these virulence-factor-related proteins. In vivo antibacterial tests showed that A_10_ controlled Xoo infection by 54.2–59.6%, which was better than thiadiazole–copper and bismerthiazol (38.1–44.9%) [117]. Based on these results, A_10_ deserves further study.

### 5.13. Thiazolidin-2-Cyanamide Derivatives and Analogs

*X. oryzae pv. Oryzae* (Xoo) is one of the main bacteria that causes rice leaf blight [118,119]. Xiang et al. [120] have been working on Xoo T3SS inhibitors (Figure 8). In 2018, they designed and synthesized a novel group of thiazolidine-2-cyanamide analogs involving the 5-phenyl-2-furan moiety (Series 1). All the molecules showed inhibitory effects against the promoter of the Harpin gene *hpa1* [120]. II-2, II-3, and II-4 showed no inhibition of the growth of Xoo, but they significantly reduced the hypersensitive response (HR) of Xoo after treatment. After Xoo was treated with these three compounds, expression of the T3SS was inhibited. For the inhibition mechanism, it was found that the three chemicals reduced the mRNA levels of representative genes related to hypersensitivity and disease, along with the regulatory genes *hrpG* and *hrpX* in the *hrp* gene cluster [120]. In vivo studies have shown that these inhibitors can alleviate the infection manifestations of rice cultivar (*Oryza sativa*) IR24 infected by Xoo.

In 2019, the authors designed and synthesized 1,3-thiazolidine-2-thione derivatives (Series 2) involving the 5-phenyl-2-furan moiety based on Series 1. All molecules tested showed inhibitory activity against the promoter activity of a harpin gene *hpa1*, among which III-7 did not interdict the bacterial growth but inhibited the expression of Xoo T3SS [118]. When studying the inhibition mechanisms, III-7 was found to reduce mRNA levels of representative genes in the hrp group with the regulatory genes *hrpG* and *hrpX*. Finally, the internal assay indicated that the molecules could decrease the symptoms of Xoo infection in *O. sativa* IR24.

Next, a series of 1,3,4-thiadiazole derivatives (Series 3) were designed and prepared based on Series 2 [119]. These compounds were found to inhibit the T3SS by reducing the expression of genes associated with the T3SS. Furthermore, similar to 1,3-thiazolidine-2-thione derivatives, this class of compounds also reduced hrpG and hrpX mRNA levels. Further studies have shown that these compounds can also alleviate Xoo infection in rice (*Oryza sativa*) [119].

Recently, a series of novel inhibitors (Series 4) were designed and prepared, and in activity tests it was found that they showed similar inhibitory activities [121]. Overall, these studies have enriched the library of compounds active against rice pathogens, and their use is expected to promote rice yields by inhibiting Xoo [121].

### 5.14. 2-Nitro-3-Arylacrylates

Ethyl 2-nitro-3-arylacrylates (Figure 8) were another group of Xoo T3SS inhibitors screened by Jiang et al. [122]. Molecules I-9, I-12, and I-13 showed good frontal inhibition of Xoo T3SS and exhibited strong inhibition against the *hpa1* promoter, with a reduction of over 80% at 100 μM. Three inhibitors at a concentration of 100 µM were co-cultured with Xoo PXO99 and did not inhibit bacterial growth [122]. At the same concentration, the three inhibitors effectively blocked Xoo T3SS hypersensitivity to tobacco. Further studies showed that I-9, I-12, and I-13 reduced the mRNA levels of the key regulatory genes *hrpG* and *hrpX* [122]. All three of these molecules have potential to alleviate the occurrence of *Xoo* in rice and *Xcc* in radish [122].

### 5.15. Benzyloxy Carbonimidoyl Dicyanides

Ma et al. [123] screened 12,000 small molecules using their T3SS inhibitor screening system for the plant pathogen *Acidovorax citrulli*. They found that a series of benzyloxy carbonimidoyl dicyanide (BCD) derivatives (Figure 8) displayed efficient inhibitory activities against the secretion of T3SS-dependent β-lactamase [123]. Among them, BCD03 observably decreased the pathogenicity of *A. citrulli* on melon seedlings and reduced hypersensitive responses in non-host *Nicotiana tabacum* induced by the pathogenic bacteria *A. citrulli*, *X. oryzae, pv. oryzae*, and *P. syringae pv. tomato* at sub-MIC concentrations. Mechanistic studies showed that BCD03 blocked the secretion of the T3SS effector protein. This suggests that BCD derivatives are a new class of T3SS inhibitors [123].

### 5.16. Other Synthetic T3SS Inhibitors

**Figure 9:**
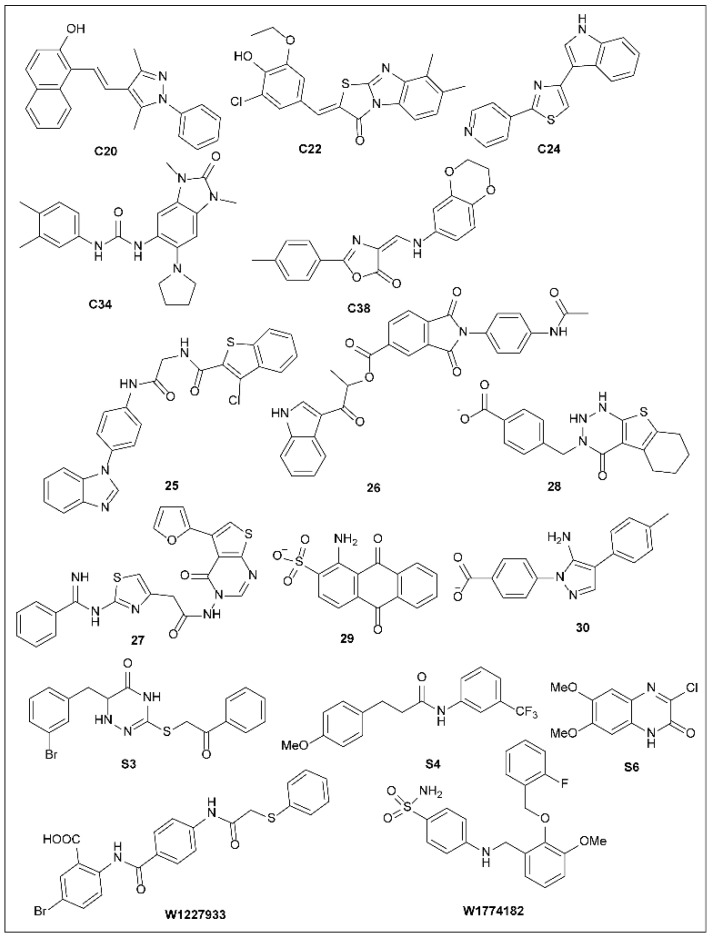
Structures of other synthetic T3SS inhibitors.

*Yersinia* spp. is a pathogenic bacterium and also uses the T3SS as a virulence factor. *pseudotuberculosis* requires a group of effector molecules called Yops, whose translocation can disrupt the original immune response of the host and lead to infection [124]. The polarization transfer process of Yops from bacteria to immune cells is complex and several conditions need to be met simultaneously, including the existence of functional T3SSs, the successful attachment of *Yersinia* to target cells, and transposable insertion into the target cell membrane. Harmon et al. [124] used high-throughput screening techniques to screen inhibitors that block Yops from entering mammalian cells. They found several inhibitors of effector protein migration that do not affect T3SS composition and effector synthesis, T3SS assembly, or effector secretion. Compound C20 reduced the adhesion of *Y. pseudotuberculosis* to the target cells. Furthermore, the inhibitors resulted in leakage of Yops into the supernatant during infection and thus decreased polarized translocation. Furthermore, C20, C22, C24, C34, and C38 (Figure 9) also inhibit ExoS-mediated cell rounding, implying that the compound targets conserved factors in *P. aeruginosa* and *Y. pseudotuberculosis* [124].

Further screening yielded inhibitors of YscN, a *Yersinia* ATPase that removes chaperones from effectors and activates the translocation process via T3SSs [125,126]. Swietnicki et al. [125] developed YscN inhibitors by computing and screening virtual three-dimensional (3D) small-molecule databases for the YscN active site model. Thirty-seven small molecules were tested for biological activity and three (**25**–**27**; Figure 9) were found to inhibit both YscN ATpase activity and YopE secretion in bacterial culture [125,126]. However, in further infection tests, these compounds showed poor inhibition because *Yersinia* still caused HeLa cells to become round in the presence of the compounds. Although the YscN inhibitors screened by the authors did not block bacterial damage to cells, this insight could be used as a springboard for future research. Bacterial ATPases have only recently been investigated as targets for antimicrobial research, and studies suggest that this enzyme is only 25% homologous to its human counterpart; therefore, concerns about drug cross-reactions are limited [125,126].

A similar development strategy could be applied to other human pathogens that contain T3SSs, including enteropathogenic *E. coli*, *S. flexneri*, *S. typhimurium*, and *Burkholderia mallei/pseudomallei* species [125]. Recently, Boonyom et al. [127] found that compound **25** interdicted the secretion of the effector protein SPI-1 of *Salmonella* T3SS at a concentration of 100 μM. The compound also attenuated the bacterial invasion of epithelial cells. They used quantitative proteomics to study the inhibitory mechanism of this compound. The results showed that compound **25** decreased the activities of the SP-1 transcription regulator InvF and the effector proteins SipA and SipC but did not block the activities of SP-1 T3SS ATPase and InvC. Further experiments are needed to elucidate the specific mechanisms by which this molecule inhibits the InvF SPI-1 regulatory protein.

Computer-aided drug design is an important way to discover new drugs. Wang et al. [82] discovered three (**28**–**30**) T3SS inhibitors using a molecular docking approach for T3SS tip protein SipD using virtual screening. In vitro antibacterial tests showed that they strongly inhibited *Salmonella* spp., including *S. enteritidis, S. typhi, S. typhimurium, S. paratyphi,* and *S. abortus equi*, with MICs ranging from 1 to 53 μg/mL [82]. In vitro cytotoxicity assays showed that they were not toxic to RAW 264.7 cells and that they could reduce the subsistence of *S. typhimurium* by 44.4, 32.5, and 52.2%, respectively, in intracellular killing assays [82]. Together, their study provides a new tool in the search for novel inhibitors of T3SS.

Mühlen et al. [128] screened six chemical libraries made up of a total of 13,360 molecules for inhibition of the translocation of the enteropathogenic *E. coli* (EPEC) effector Tir (translocated intim receptor). Three T3SS inhibitors (**S3**, **S4**, and **S6**) were found to display dose-dependent inhibition of Tir translocation. Cell experimental studies showed that these three compounds weakened the intimate attachment between the EPEC strain E2348/69 pP*_gapdh_amCyan* and HEP-2 cells [128]. None of the three inhibitors exhibited an effect on bacterial motility. However, hemolysis assays showed differing activities of the three compounds with regard to hemolysis inhibition. S3 had no inhibitory effect on T3SS-dependent hemolysis of sheep red blood cells (RBCs), whereas S4 and S6 strongly blocked this effect by 20% and 10%, respectively, compared with DMSO-treated cells [128]. Gaussia luciferase reporter gene assays in *E. coli* K12 (C600) and *C. rodentium* (DBS100) strains confirmed that the three inhibitors could not be induced to produce Shiga toxin. Their study identified three safe and efficient T3SS inhibitors to treat EPEC and EHEC infected diseases.

Grishin et al. [129] identified two *Chlamydial* T3SS inhibitors, W1227933 and 1774182, through virtual screening against T3SS ATPase. Treatment with W1227933 and 1774182 of McCoy cells infected with *C. trachomatis* showed dose-dependent inhibitory effects [129], and the inclusion of *Chlamydia* was much smaller than with no treatment. Furthermore, tests on the cytotoxicity of the two compounds showed that the cell survival rate was 95–100% at 50 µM and 70–80% at 100 µM, indicating that the two inhibitors were less toxic to living cells [129]. Both inhibitors inhibited the transport of the T3SS-mediated effector protein to the *Chlamydia* inclusion membrane at 25 µM. Although these two compounds were obtained by virtual screening of SctN active sites, it is not yet clear whether they have SctN inhibitory activity.

## 6. Naturally Occurring T3SS Inhibitors

Natural molecules from plants, animals, and microorganisms are important sources of leads for drug discovery and development. After extensive screening, several research teams have found natural products with inhibitory activities against the T3SS, enriching the types and quantities of T3SS inhibitors.

### 6.1. Caminosides

**Figure 10:**
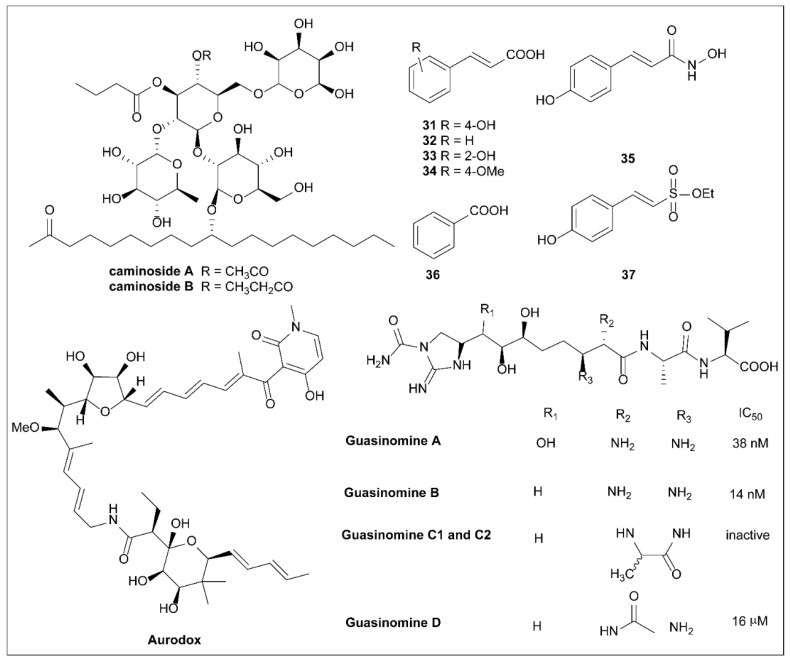
Structures of Caminoside A, Caminoside B, **31**–**37**, Aurodox, and Guasinomines.

Although most strains of *E. coli* are harmless to humans, several can cause illness [16]. For example, infant diarrhea in underdeveloped countries is often caused by EPEC infection, which causes diarrhea by infecting human intestinal epithelial cells [130,131]. Unlike other pathogenic bacteria, EPEC does not infect host cells but uses T3SSs to deliver bacterial effectors to host cells [130]. In 2002, Linington et al. [132] isolated the complex glycolipid Caminoside A from a marine sponge *Caminus sphaeroconiia* (Figure 10).

In a biological test designed to screen for inhibitors of T3SS, Caminoside A (**46**) was found to reduce EspB but not EspC secretion and to block the pathogenicity of EPEC by reducing its virulence without killing the bacterium [133]. The discovery of Caminoside A represents the first natural T3SS inhibitor (IC_50_ = 20 µM).

To confirm that Caminoside A inhibited T3SS but not EPEC growth, conventional studies of Caminoside A against a group of human and plant pathogens were performed. The results indicated that Caminoside A showed good inhibitory activity against methicillin-resistant *S. aureus* (MIC = 12 µg/mL) and vancomycin-resistant Enterococcus (MIC = 12 µg/mL) in vitro but no activity against *E. coli* (MIC > 100 µg/mL). As an analog of Caminoside A, Caminoside B was also reported by Linington et al. and showed similar inhibitory activity against the T3SS [133].

### 6.2. Plant Phenolic Compounds

Plants synthesize phenolic compounds as secondary metabolites. To defend themselves against pathogen invasion, plants have developed a systematized acquired resistance mechanism [134]. As a plant responds to bacterial pathogens, it recognizes the T3SS effectors or their actions and initiates an array of defense responses, which can include programmed cell death when being attacked. In their study, Li et al. [135] showed that p-coumaric acid (PCA, **31**; Figure 10) repressed the expression of the T3SS genes of the plant pathogen *Dickeya dadantii*, suggesting that plants can already resist pathogenic attack by managing the expression of the T3SS.

The HrpX/Y two-component system is the core regulatory factor of the T3SS, and PCA inhibits the expression of T3SS regulatory genes by regulating it. Furthermore, trans cinnamic acid (**32**) and o-coumaric acid (**33**) can also affect the RsmB–RsmA pathway and induce the expression of the T3SS gene *hrpA* in *D. dadantii.* Subsequent studies found that **31** was converted to trans-4-hydroxycinnamic hydroxamic acid (**35**), which was eight times more potent against T3SS in *D. dadantii*.

As inhibitor **35** showed potent activity, further experiments were performed to confirm the mechanism of action. Hydroxamic acid **52** was found to reduce the transcription levels of *hrp*S and *hrp*L by inhibiting HrpY phosphorylation. It was confirmed that compound **35** inhibited *hrp*L at the post-transcriptional level by lowering the levels of the RNAs that regulate small RNA RsmB, thereby influencing the RsmB–RsmA regulatory pathway. Therefore, it was concluded that compound **35** inhibited *hrp*L transcription and mRNA stability, resulting in decreased expression of HrpL regulatory genes, for example, hrpA and *hrp*N. Compound **35** is therefore the first T3SS inhibitor that has been found to be active against the soft rot pathogen *D. dadantii* 3937, and it has this effect by affecting both the transcription process and the post-transcription pathway [136]. Fire blight is a serious disease of the Rose family, usually caused by *E. amylovora* [137]. The T3SS is an indispensable virulence factor of *E. amylovor*, used by *E. amylovor* to invade host cells. The ability of phenolic compounds to alter T3SS expression aroused the interest of Li et al. [136]. They used a constructed GFP reporter gene and high-throughput flow cytometry to screen a library of phenolic compounds. Compounds **34** and **36** inhibit T3SS activity by affecting the expression of pilus and trans-2-(4-hydroxyphenyl)-vinyl sulfonate (EHPES, **54**), which are inducers of T3SS. TMCA, BA, and EHPES all affect the HrpS–HrpL pathway, which alters the expression of T3SS. TMCA also alters T3SS expression by affecting the *rsm*B_Ea_–RsmA_Ea_ system [137]. Finally, TMCA and BA were found to attenuate tobacco hypersensitivity by inhibiting the T3SS of *E. amylovora* [137] (Figure 10). Recently, T3SS inhibitors with similar structures have been reported [3,138].

### 6.3. Aurodox

In 1973, Berger et al. [139] isolated a new antibiotic named X-5108 (Aurodox; Figure 10) from *Streptomyces goldiniensis*, and it was originally characterized by UV, IR, and NMR spectra and optical rotation. Interestingly, Aurodox showed different biological activities in vitro and in vivo, mainly inhibiting Gram-positive bacteria in vitro, while effectively inhibiting *Streptococcus pyogenes* infections in mice and significantly promoting poultry growth in vivo. It showed low toxicity in mice, with an LD_50_ of >1 g/kg sbc and >4 g/kg p.o [139]. Chinali [140] developed a series of Aurodox derivatives and tested their effects on elongation factor Tu (EF-Tu), followed by a structure–activity study [16].

In 2011, Kimura et al. [141] established a T3SS-mediated hemolysis screening system for EPEC, which they used to identify Aurodox, a specific T3SS inhibitor, as a mediator of hemolysis by *Streptomyces* sp. Aurodox can inhibit the T3SS and inhibits hemolysis with an IC_50_ value of 1.5 μg/mL in the absence of inhibiting pathogen growth in the liquid medium. The study also confirmed that Aurodox specifically inhibited the secretion of T3SS secreted proteins, including EspB, EspF, and Map, but did not affect the expression of the housekeeping protein GroEL. In addition, an animal study revealed that, unlike the control, mice infected with a fatal dose of *C. rodentium* survived after Aurodox injections. Therefore, the study by Kimura et al. [141] is the first to directly demonstrate that Aurodox represents a potential new class of anti-infective agents.

In recent studies, new findings from McHugh et al. [142] suggest that Aurodox down-regulates enterpathogenicity and EHEC T3SS expression. Using transcriptome analysis, they confirmed that Aurodox inhibited T3SS expression at the transcriptional level by inhibiting the main regulator Ler. This experiment suggests that Aurodox does not directly inhibit T3SS itself but acts on Ler upstream of it [142]. Finally, their experiments yielded different results for some conventional antibiotics, in that, although RecA is essential for *Shiga* toxin production, Aurodox does not induce RecA expression. These properties suggest that Aurodox is a promising treatment for these infections and an excellent antitoxic therapy [142]. In addition, the interesting biological activity and complex structure of Aurodox attracted synthetic chemists to achieve its total synthesis [143,144].

### 6.4. Guasinomines

Iwatsuki et al. [145,146] isolated six guadinomines (Figure 10) using EPEC-mediated hemolysis to screen product extracts from *Streptomyces* sp. K01-0509 in 2008. Guadinomines A and B showed the strongest inhibitory activity against T3SS, with IC_50_ values of 38 nM and 14 nM, respectively, and Guadinomine D showed moderate activity (IC_50_ = 16 μM), whereas guadinomines C1 and C2 had no activity. Due to the low yields of these compounds in culture, further research was hindered and the mechanism of action of guanidine compounds has not been explored. Hirose et al. [147] reported the total asymmetric synthesis of guadinomines B and C2 with 33 linear steps and confirmed their absolute configurations. In 2012, Holmes et al. [148] analyzed and reported the biosynthetic pathway of Guadinomine A. Compared to Guadinomine B, Guadinomine D has an amide as the R2 substituent, and its inhibitory activity is 1000 times lower. Although Guadinomines do not suppress bacterial growth, Iwatsuki et al. [145] discovered that Guadinomine B is cytotoxic to Jukat cells, with a concentration 100 times higher than the IC_50_ value. Therefore, further study of Guadinomine B may provide more beneficial results.

### 6.5. Piericidins

**Figure 11:**
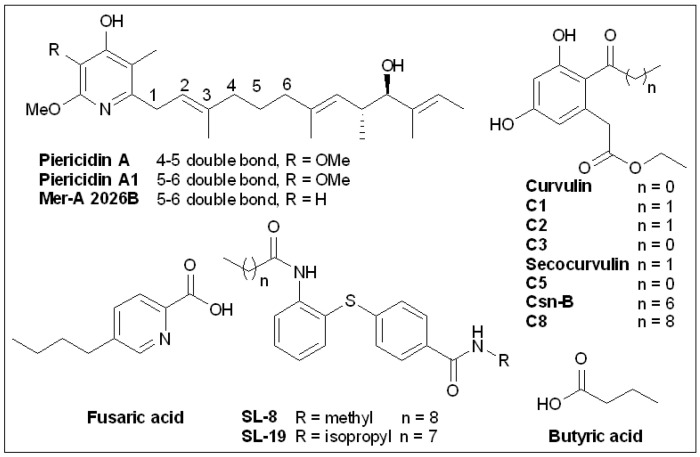
Structures of piericidins, Csn-B and derivatives, butyric acid, and fusaric acid and derivatives.

During a screen for new insecticides among metabolites of microorganisms, Tamura et al. [149] isolated the pyridine derivative Piericidin A (Figure 11) from *Streptomyces* sp. 16–22 in 1963, but the full structure was not elucidated until 1965 [150]. The researchers analyzed the composition of microbes in soil samples from the Chiba prefecture in Japan and tested them for toxicity to various larvae. Among them, *Streptomyces* sp. 16–22 displayed the highest toxicity. Tamura et al. [149] obtained Piericidin A through bioactivity-oriented separation, confirmed its structure, and tested its chemical properties. The same study showed that Piericidin A was not highly cytotoxic against Gram-negative bacteria, such as *E. coli* and *X. oryzae*.

In 1966, studies by Hall et al. [151] elucidated Piericidin A as a new mitochondrial electron transport inhibitor. Piericidin A showed strong inhibitory activity against DPNH oxidase and showed a complete inhibitory effect at 0.036 μmol/mg of mitochondrial protein. However, inhibition of succinic acid oxidase was weak, with an inhibition rate of 50% at 0.33 nmol/mg protein and 80% at 2.0 μmol/mg protein [151].

In 2014, Duncan et al. [110] screened two natural T3SS inhibitors from marine actinomycetes and preliminarily characterized them. Although bacterial extracts containing Piericidin A1 and Mer-A 2026B (a piericidin derivative) were not toxic to mammalian cells, they inhibited *Y. pseudotuberculosis* in HEK293T cells by inducing t3S-dependent activation of host transcription factor NF-κB. Since *Yersinia* T3SS must function normally to trigger NF-κB activation, these results suggest that Piericidin A1 and MER-A 2026B inhibit the T3SS. Purified Piericidin A1 and Mer A-2026B showed dose-dependent inhibition of the translocation of the T3SS effector protein YopM (a 75% reduction at 71 µm) of *Y. pseudotuberculosis* in CHO cells [97]. Unlike other antibiotics, neither inhibitor blocks the growth of bacteria in vitro.

When *Yersinia* was cultured without host cells under T3SS induction, Mer-A 2026B and Piericidin A1 also showed similar or better activities against the secretion of T3SS cargo than the inhibitors reported above (MBX-1641 and Aurodox) [110].

These results suggest that Mer-A 2026B and Piericidin A1 do not inhibit T3SSs by blocking bacterial invasion of host cells, but block early synthesis of T3SSs, such as the assembly of T3SS needles. Initially, researchers defined Piericidins as inhibitors of the electron transport chain complex I in the mitochondria of some bacteria [151]. However, Morgan et al. [152] found that Piericidin A1 did not change *Yersinia* membrane potential or inhibit proton-driven flagellar movement, suggesting that inhibition of Piericidin and *Yersinia* T3SS was independent of complex I. In contrast, piericidin A1 decreased the number of T3SS acicular complexes on bacterial surfaces and blocked T3SS translocation and effector protein secretion [152].

Other experiments confirmed that Piericidin A1 reduced the abundance of the high-order YscF needle subunit complex and hindered YscF needle assembly [152]. T3SS expression in *Yersinia* is positively regulated by T3S secretion activity, but the secretion blockage caused by Piericidin A1 is not accompanied by a decrease in T3SS gene expression, suggesting that Piericidin A1 may target the T3SS regulatory circuit. However, piericidin A1 inhibits effector protein secretion even when the T3SS modulators YopK, YopD, or YopN are absent. It is surprising that piericidin A1 also interdicted the T3SS of *Y. enterocolitica* Ysc without blocking the Ysa T3SS of the SPI-1 family Ysa in *Y. enterocolitica* or the Ysc family T3SS in *P. aeruginosa*. The above results indicated that Piericidin A1 specifically inhibited the assembly of the *Yersinia* Ysc T3SS needle [152].

### 6.6. Cytosporone B and Derivatives

In 2002, Brady et al. [153] isolated cytosporone B (Csn-B; Figure 11), along with four new octaketides, from *Dothiorella sp*. HTF3, an endophytic fungus (Figure 9). Zhan et al. [154] reported for the first time that Csn-B is a natural Nur77 agonist through in-depth experiments. Furthermore, Csn-B can also delay the growth of xenograft tumors because it can induce Nur77 expression, transfer Nur77 to the mitochondria, cause the release of cytochrome C, and induce cell apoptosis [154,155,156].

On account of the rich physiological activity and easy-to-modify structure of Csn-B, structure–activity studies have been conducted on Csn-B. A series of Csn-B derivatives were synthesized and screened for activity as T3SS inhibitors in *S. enterica* Serovar Typhimurium [157]. In vitro, Csn-B and several analogs did not affect the secretion of flagellin FliC but strongly inhibited the secretion of pathogenic island 1 (SPI-1)-related effector proteins of *Salmonella* (IC_50_ = 6.25 μM). Csn-B, **C5**, and secocurvulin had no significant effects on bacterial growth but strongly inhibited SPI-1-mediated invasion of HeLa cells. The nucleoid proteins Hha and H-NS bind to the promoters of the SP-1 regulatory genes *hil*D, *hil*C, and *rts*A, inhibiting the expression of these promoters and thus regulating the expression of the SP-1 device and effector genes. The results showed that Csn-B up-regulates the transcription of *hha* and *hns*, suggesting that Csn-B could affect effector secretion through the Hha–H-NS regulatory pathway. Overall, this study was the first to report that Csn-B is an effective SPI-1 inhibitor and can contribute to the development of a treatment for drug-resistant *Salmonella* [157]. Due to the various bioactivities of Csn-B, several synthetic routes have been developed for synthesis of this compound [158,159,160,161,162,163].

### 6.7. Butyric Acid

In 1964, Bohnhoff et al. [164] found that fresh fecal buffer suspensions taken from the large intestine of healthy mice inhibited the growth of *S. enteritidis* in vitro, with material taken from the cecum and transverse colon being the most effective.

These materials still have antibacterial activity after thermal sterilization or filtration sterilization. Acetic and butyric acids (Figure 11), which inhibit *Salmonella* concentrations, were isolated from these materials under in vitro conditions [164]. As these experiments were conducted before T3SS was reported, the authors did consider T3SS inhibition as a mode of action [78]. Butyric acid is the fermentation product or the end product of the intestinal microbiota [165]. In the human intestine, the concentration of butyric acid is generally 10–20 mM [166]. The main energy of colon cell metabolism comes from butyric acid, and the ability of colon cells to absorb and use sodium butyric acid is an indicator of human health [166]. However, Nakanishi et al. [167] found that different concentrations of short-chain fatty acids (SCFAs) had different effects on EHEC. At low concentrations, SCFAs significantly enhanced the expression of virulence genes required for cell adhesion, adhesion induction, and the induction of attaching and effacing (A/E) lesions, which facilitated the EHEC invasion of cells. However, high concentrations inhibited the growth of EHEC. When different SCFAs were selected, butyrate significantly increased the expression of these virulence-related genes, even at concentrations as low as 1.25 mM. However, even at high concentrations of 40 mM, acetate and propionate showed only weak effects [167]. Butyrate increased the promoter activity of the *LEE1* operon, which encodes a global regulator of the LEE gene Ler. This enhancement depended on a regulator, PchA. Butyrate sensing was completely lost by deletion of *Irp*, the gene for the leucine-responsive regulatory protein, Lrp [167]. Lrp does not control gene expression from one organism to another in the same pattern. From the above results, it can be concluded that butyrate inhibits the T3SSs of some microorganisms, while it activates the T3SSs of others [78,167,168,169,170]. For example, there are 90% homologous sequences in pathogenic islands of LEE in EPEC and *C. Rodentium*, making their T3SSs similar. *C. rodentium* is often used as an EPEC-infected mouse model. However, the T3SS regulator Lrp is not encoded by LEE, so Lrp activation has opposite reactions in the two bacteria [169,170], with activation of Lrp up-regulating expression of LEE in EPEC but showing down-regulation in *C. rodentium* [167,170]. The effect of probiotics on infection is the main research focus regarding SCFAs as regulatory factors of T3SSs [165,168,170]. Changes in the abundance of SCFA-producing bacteria in the intestine result in different proportions of SCFAs and these may have differing effects on the various intestinal pathogens, with some improving infection and others exacerbating infection. As probiotics are widely used, this area needs further research.

### 6.8. Fusaric Acid and Derivatives

Fusarium acid (FA; Figure 11) is produced by *Fusarium oxysporum* and is an important virulence factor for various plant diseases, such as Fusarium wilt in banana, tomato, and cotton and heavy decline disease in grape [171,172,173]. Studies have found that FA has an inhibitory effect on microorganisms [171,173]. In 2014, a small-compound library was screened for inhibitory activity against T3SSs, and FA was found to be a potential inhibitor of *S. enterica* T3SSs [174]. In this experiment, with solvent as a control, FA dramatically blocked the secretion of SipA/B/C/D, the effector of SP-1. However, FA does not affect the flagellar secretion of flagellin FliC. Furthermore, within a certain dose range, the inhibitory effect of FA on SPI-1 increased with increasing dose, with an IC_50_ value of 53.5 μM. Next, the study showed that the effect of FA on the growth of *S. enterica* Serovar Typhimurium was not the cause of its inhibitory effect on SPI-1 [174]. Since FA inhibited the secretion of SPI-1 effectors, the researchers wanted to know whether this compound had a blocking effect on *Salmonella* invasion of host cells [174].

The MTT results showed that there was no significant change in the cell viability of HeLa cells treated with FA at different concentrations for 72 h. With gentamicin protection, FA significantly inhibited *Salmonella* invasion of HeLa cells compared to the solvent control, using a known SPI-1 inhibitor Csn-B as a positive control (*p* < 0.001) [157]. Interestingly, FA expression in this study differed from the reported T3SS inhibitors in the mechanism of action, so the target protein still needs to be identified. For example, the mechanism by which FA affects proteins such as SIA/InvF could be investigated [174].

To find T3SS inhibitors with better activity, Song et al. [175] optimized the structure of FA and synthesized a series of diphenyl sulfide compounds through the scaffold hopping method. The T3SS inhibitory activities of these 22 derivatives were screened, and SL-8 and SL-19 were found to show stronger T3SS inhibitory activity and anti-cell infection activity compared to the positive control FA [175]. SDS-PAGE showed that the inhibition rates of SL-8 and SL-19 against the T3SS effector protein SipC reached 90.8% and 89.9% in vitro at a concentration of 100 μM, respectively. This inhibition was dose-dependent. Western blot analysis showed that the IC_50_ values of SL-8 and SL-19 in SipC were 14.6 μM and 6.1 μM, respectively, both being lower than that of FA (53.5 μM). When SL-8 and SL-19 were co-cultured with bacteria, it was found that neither of them inhibited bacterial growth. The cell infection test showed that SL-8 and SL-9 could effectively inhibit bacterial infection of Caco-2 cells [175]. To investigate the inhibition mechanism, Western blot analysis of SipC contents in bacteria treated with SL-8 and SL-19 showed that total SipC contents and cytoplasm contents were similar to those of the controls, but there were decreased SipC contents in the broth and increased SipC contents in cell debri. These results confirmed that these derivatives inhibit SipC secretion but not transcription and translation [175]. In conclusion, they synthesized FA derivatives and obtained compounds with better T3SS inhibition activities than FA, which provided references for subsequent studies.

### 6.9. (-)-Hopeaphenol

**Figure 12:**
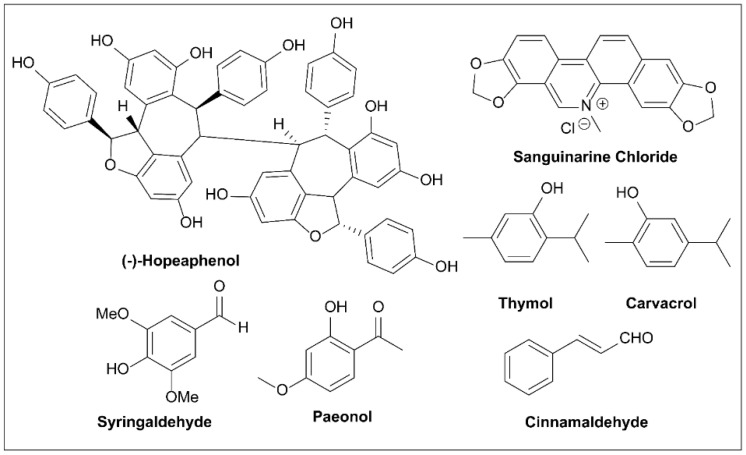
Structures of Hopeaphenol, Sanguinarine Chloride, Thymol, Carvacrol, paeonol, and Cinnamaldehyde.

In 2013, Zetterström et al. [176] discovered resveratrol tetramer (-)-hopeaphenol (Figure 12) as a T3SS inhibitor of *Y. pseudotuberculosis* from a natural product library. Furthermore, other studies explored antioxidant properties and the inhibition of the SARS-CoV-2 protease of (-)-hopeaphenol [177]. Eloffson et al. studied the inhibitory activity of (-)-hopeaphenol against multiple pathogenic bacterial T3SSs, including those of *Y. pseudotuberculosis, P. aeruginosa,* and *C. trachomatis* [176]. First, they tested the activity of (-)-hopeaphenol using a YopE reporter gene assay and a YopH phosphatase assay. The results showed that the activity of (-)-hopeaphenol was dose-dependent, with IC_50_ values of 6.6 µM and 3.3 µM for the YopE reporter gene test and the YopH phosphatase test, respectively.

Furthermore, when the concentration was increased to 100 µM, (-)-hopeaphenol had no or a finite impact on bacterial growth [176]. YPIII (pIB102) wild-type bacteria were incubated with seven concentrations of (-)-hopeaphenol at 26 °C for 1 h, followed by 37 °C for 3 h; then, Western blotting was used to analyze effector proteins in the total culture medium and supernatant. The results showed that both protein YopD secretion and transporter expression were dose-dependent.

The decreased expression of YopD was detected at all concentrations, but the secretion of YopD was completely blocked when the concentration was greater than 13 μM. These results suggest that (-)-hopeaphenol may directly affect the secretion mechanism, rather than cause T3SS gene transcription attenuation in *E. coli* [176]. In addition, the authors demonstrated that (-)-hopeaphenol is an irreversible inhibitor of T3SSs through co-culture experiments. The *P. aeruginosa* effector protein ExoS is like the *Y. pseudotuberculosis* effector protein YopE in structure; therefore, *P. aeruginosa* was co-cultured with (-)-hopeaphenol, and Western blot analysis showed that, compared with the control, (-)-hopeaphenol reduced the expression and secretion of the effector protein ExoS in bacteria. ExoS secretion was reduced at 10 and 20 µM and was completely blocked at 50 and 100 µM [176]. In 2014, Davis et al. [178] identified three known resveratrol tetramers, (-)-hopeaphenol, vatalbinoside A, and vaticanol B, from the leaves of *Anisoptera thurifera* and *Anisoptera polyandra.* Their experiments showed that (-)-hopeaphenol, vatalbinoside A, and vaticanol B inhibited YopE, with IC_50_ values of 8.8, 12.5, and 9.9 μM, respectively, in a luminescent reporter gene assay, and with IC_50_ values of 2.9, 4.5, and 3.3 μM, respectively, in an enzyme-based YopH assay [178]. The results implied that the tetramers could effectively block T3SSs in *Yersinia*. More importantly, vatalbinoside A can be converted to (-)-hopeaphenol by endogenous leaf enzymes. This transformation can improve the yield of (-)-hopeaphenol, which is helpful for subsequent bioactivity studies [138,178,179].

### 6.10. Sanguinarine Chloride

Babich et al. [180] isolated sanguinarine chloride (Figure 12), a natural alkaloid, from *Sanguinaria canadensis*. In the 1970s and 1980s, researchers discovered that sanguinarine chloride has anti-inflammatory properties and considered it a potential treatment for gingivitis. It is also being studied as a chemotherapeutic agent [180,181]. In 2018, Zhang et al. [182] confirmed the inhibitory activity of sanguinarine chloride against the T3SS of *S. enterica* Serovar Typhimurium. They found that sanguinarine chloride could effectively inhibit *S. enterica* Serovar Typhimurium secreting effector proteins into HeLa cells.

Most HeLa cells showed blue fluorescence, indicating invasion of HeLa cells, and the MOI reached 50 when SL1344 with β-lactamase fusion protein was transfected without sanguinarine chloride [182]. In contrast, most HeLa cells showed green fluorescence in the presence of sanguinarine chloride (5 μM), indicating a significant reduction in translocation of the β-lactamase reporter gene. In addition, the cytotoxicity of sanguinarine chloride in HeLa cells was determined by an LDH release assay. The results showed that 10 μM of sanguinarine chloride had slight cytotoxicity, whereas 20 μM of sanguinarine chloride caused damage to HeLa cell membranes [182]. The sanguinarine chloride was found to inhibit the transcription of downstream genes induced by HilA. Animal studies showed that sanguinarine chloride exhibited animal toxicity even at a dose of 20 mg/kg [182]. In the future, it should be modified to reduce its drug toxicity.

### 6.11. Thymol and Carbvacrol

Thymol structurally belongs to the substituted phenol class and is an essential oil of the genus *Thyme* [183,184]. Recently, Zhang et al. [184] demonstrated that thymol (Figure 12) can effectively inhibit T3SS-1 of *S. typhim*. They found that at 0.4 mM, thymol did not damage HeLa cell membranes and did not affect the growth of the *S. typhimurium* strain SL1344. Considering that a high concentration of thymol can cause potential complications, they used 0.2 mM or a lower concentration in the next assay [184]. To test the protective effect of thymol on infected mice, they conducted animal experiments and found that 80% of the mice infected with wild-type bacteria died on day 6 and that all died on day 7. When the infected mice were injected with thymol at a dose of 50 mg/kg, three times a day until day 6, no infected mice died, 80% of the mice survived until day 7, and 70% until day 9. These results showed that thymol could easily reduce the mortality of infected mice, suggesting that the compound could control *Salmonella* infection [184].

*S. typhimurium* is one of the main bacteria that cause gastroenteritis in humans [184]. Pork is one of the main transmission routes of *S. typhimurium*, and Giovagnoni et al. [185] believe that the use of bioactive compounds as feed additives can help control the spread of *S. typhimurium*. Recently, they evaluated the efficacy of sublethal concentrations of thymol and carvacrol in blocking *S. typhimurium* infection of Caco-2 cells, focusing on the maintenance of the epithelial barrier changes in *Salmonella* virulence genes. The results showed that thymol and carvacrol had a protective effect on the integrity of the intestinal monolayer, while improving transepithelial resistance and bacterial translocation [185]. A real-time PCR study found that thymol and carvacrol significantly down-regulated the major virulence genes (*hilA, prgH, invA, sipA, sipC, sipD, sopB,* and *sopE2*) in *Salmonella*. The results show that thymol and carvacrol may help prevent and treat *S. typhimurium* in pigs [185].

### 6.12. Syringaldehyde

Syringaldehyde (Figure 12) is a substituted benzaldehyde, derived from the stems of *Hibiscus taiwanensis* (Malvaceae), which has hypoglycemic effects [186]. Studies have shown that syringaldehyde can significantly reduce postprandial blood glucose in rats without modifying insulin, suggesting that syringaldehyde may reduce blood glucose in diabetic rats by increasing glucose utilization [186].

Based on previous experimental methods, Lv et al. [187] confirmed that syringaldehyde was an effective inhibitor of *S. typhimurium* T3SS. When the concentration of syringaldehyde was 0.18 mM, bacterial growth was not affected, but the expression of important effector proteins (SipA, SipB, and SipC) was inhibited [187]. Furthermore, syringaldehyde reduced mortality (40%) and bacterial load in mice infected with *S. typhimurium,* reduced cecal injury and systemic inflammation, and provided systemic protection against infection [187]. Based on these results, syringaldehyde could be a potential lead compound against *S. typhimurium* infections because it significantly inhibited T3SS activity [187].

### 6.13. Paeonol

Paeonol refers to an active ingredient extracted from the dried root bark of *Paeonia moutan* Sim, a plant in the buttercup family (Figure 12). Studies have revealed that paeonol has analgesic, anti-inflammatory, antipyretic, and antiallergic effects [188,189]. In 2020, based on the previous screening system, Lv et al. [189] found that paeonol could effectively interdict intracellular translocation of *S. typhimurium* T3SS effector protein SipA at 0.19 mM and within a concentration range of 0.048 to 0.76 mM. Furthermore, paeonol had no cytotoxicity against HeLa cells, and therefore the effects of paeonol on the growth of *S. typhimurium* were further analyzed. Paeonol did not affect the growth of *S. typhimurium* in a concentration range of 0.048 to 0.76 mM, and DMSO did not cause harm to *S. typhimurium* at corresponding volumes. These results indicate that paeonol interdicts T3SSs by blocking the translocation of the effector protein SipA. Most importantly, paeonol is not cytotoxic and does not inhibit bacterial growth. In addition, paeonol significantly reduced levels of *S. typhimurium*-mediated cell damage and invasion. Animal experiments confirmed that paeonol had a comprehensive protective effect on *S. typhimurium*-infected mouse models. Preliminary mechanistic exploration experiments showed that paeonol reduced the transcription level of the *hilA* gene in the SPI-1 regulatory pathway, thus inhibiting the expression of the effector protein [190]. The work implied that paeonol could be a potential chemical to treat infections caused by *Salmonella*.

### 6.14. Cinnamaldehyde

Cinnamon is a seasoning often used in Chinese cooking, from which an effective antibacterial essential oil, cinnamaldehyde, can be isolated, which is also the main contributor to cinnamon flavor [191] (Figure 12). Studies by Subash Babu et al. [192] showed that cinnamaldehyde significantly reduced blood glucose and lipid levels in STZ-induced diabetic rats. To find a potent T3SS inhibitor, Liu et al. [193] screened a variety of natural products for inhibitory activity against T3SSs. Their results showed that cinnamaldehyde inhibited *Salmonella* pathogenicity island 1 (SPI-1) by blocking the secretion of several SPI-1 effector proteins and strongly inhibited SPI-1-mediated *Salmonella* invasion of HeLa cells. Further studies revealed that the mechanism by which cinnamaldehyde inhibits SPI-1 involves the affection of multiple SPI-1 regulatory genes and significant reduction of the transcription of some SPI-1 genes, such as SipA and SipB [193]. Animal studies have confirmed that cinnamaldehyde can effectively reduce mortality and pathological damage in infected mice. Their study identified a potent T3SS inhibitor, cinnamaldehyde, which works by affecting the transcription of major regulatory genes to reduce the expression of SP-1 effector proteins [193].

### 6.15. Flavonoids

**Figure 13:**
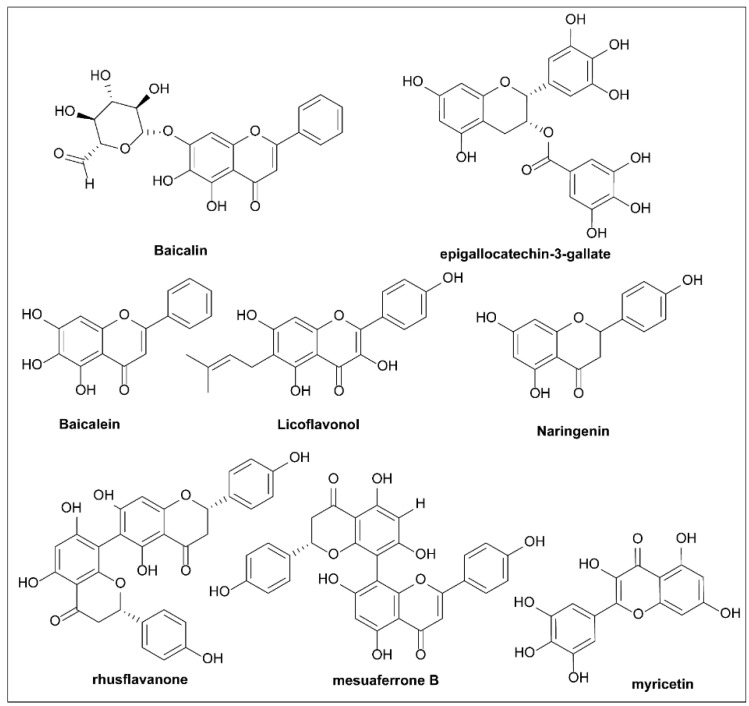
Structures of flavonoids.

Flavonoids are a type of secondary plant metabolite with a polyphenol structure. They are widely distributed in various plants and are important natural products (Figure 13). They have rich biological activities and are widely used in the treatment of various diseases, such as cancer, Alzheimer’s disease (AD), and atherosclerosis [194]. Increasingly, because of their reported ability to fight pathogens, flavonoids have become targets of anti-infective drug development [195]. In recent years, various flavonoids have been reported to exhibit inhibitory activity against T3SSs.

As a glycoside, baicalin observably reduced the body weight, serum levels of TNFα, IL-6, and LDH, and caecum bacterial burdens of mice infected with *S. typhimurium* [196]. Histological examination confirmed that baicalin can alleviate the cecal injury caused by *S. typhimurium* infection in mice. The MIC and MBC of baicalein against *S. typhimurium* were 64 μg/mL, and >128 μg/mL, respectively. The pretreatment of Caco-2 cells or *S. typhimurium* with baicalin dramatically blocked Caco-2 cell invasion by *S. typhimurium* in a dose-dependent manner [196]. Further studies showed that baicalin inhibited T3SSs by inhibiting sopB, sopE, and sopE2 transcription levels. Therefore, it is a promising drug to prevent *S. typhimurium* infection by inhibiting bacterial virulence and regulating host response [196].

In 2016, Tsou et al. [197] identified a different mechanism by which baicalin targets SPI-1 and translocation enzymes of *S. typhimurium* T3SS to inhibit bacterial invasion of epithelial cells. Interestingly, flavonoids, such as quercetin, which have structures similar to baicalin and are found in other traditional Chinese medicines (TCMs), also inhibit SPI-1 of the T3SS and block the invasion of *S. typhimurium* [197]. These results suggest that specific active ingredients from TCMs can interfere with key virulence pathways of bacteria and reveal a previously unappreciated direction in the development of anti-infective drugs [197].

Furthermore, Tsou et al. also found that epigallocatechin-3-gallate from green tea has inhibitory activity against the T3SS protein effector of *S. typhimurium* and could protect host cells from bacterial invasion [198]. These results suggest that the addition of plant metabolites to food can attenuate bacterial virulence and prevent the infection of host cells [198]. Shen et al. screened the anti-T3SS activity of 20 prenylated flavonoids and found that several analogs could strongly inhibit the secretion of SPI-1-related efferent proteins from *S. typhimurium* but did not block the growth of bacteria and the secretion of flagellin FliC [199]. However, licoflavonol regulates SicA/InvF gene transcription and SipC transport, inhibiting the secretion of the SPI-1 effector protein [199]. These results indicate the licoflavonol might be an important compound for the development of new antibiotics [199].

Using cDNA microarray technology, Patil et al. studied the effects of naringenin treatment on *S. typhimurium* LT2. The results showed that it could significantly inhibit 24 genes in SPI-1 of *S. typhimurium* LT2 and that it down-regulated the expression of 17 genes related to the flagella and motility, also showing specificity [200]. Furthermore, phenotypic assays supported the results of microarray analysis [200], and naringin was found to inhibit SPI-1 in a pstS/hilD-dependent manner. In conclusion, naringenin can reduce the virulence of *S. typhimurium* and promote cell viability [200].

Two bioflavonoids were isolated from the ethyl acetate extract of the *Mesua ferrea* flower by Shen et al. and screened for their inhibitory activities against the T3SS [201]. The authors found that the biflavonoids rhusflavanone and mesuaferrone B could block the secretion of SPI-1 effector proteins (SipA, B, C, and D) and that they did not affect bacterial growth [201]. Myricetin is a flavonoid compound with antioxidant and anti-tumor properties. Furthermore, in vitro studies have shown that myricetin can modify LDL cholesterol at high concentrations and increase its uptake by white blood cells [202]. High myricetin consumption has also been shown to reduce the risk of prostate and pancreatic cancer [203,204]. Recently, Wang et al. studied the inhibitory effect of myricetin on the T3SS [205]. The preliminary study showed that it exhibited inhibitory activity with respect to translocation of the SPI-1 effector (SipA and SipB) at a concentration of 4 μg/mL [205], whereas it did not cause growth arrest of *S. Typhimurium* SL1344. Additional experiments showed that myricetin significantly inhibited SipC expression at a concentration of 1 μg/mL. Further studies on the inhibitory mechanism showed that myricetin reduced the levels of hilA, sopA, sicA, and prgH, thus inhibiting the expression of the SP-1 effector protein. Bacterial infection of HeLa cells treated with myricetin was further studied, and at a concentration of 8 μg/mL it could significantly inhibit *Salmonella* invasion of HeLa cells without resulting in cytotoxicity [205]. 

### 6.16. Limonoids

**Figure 14:**
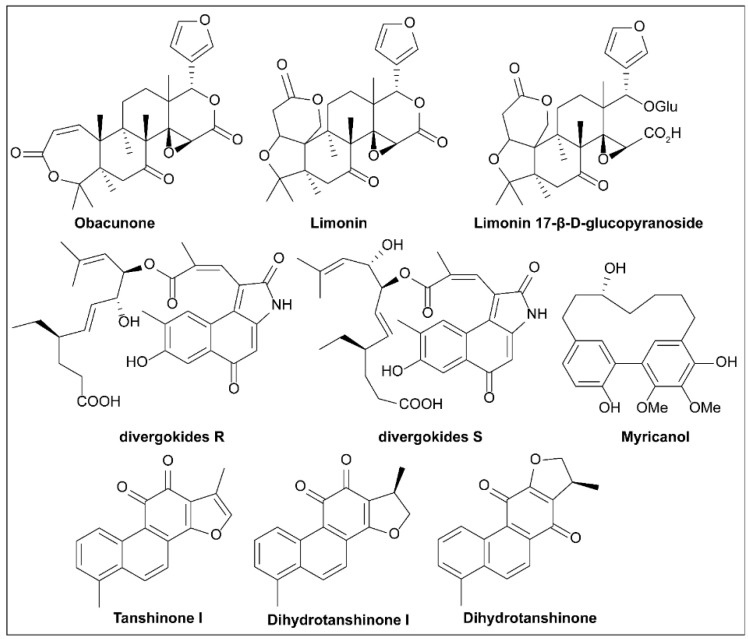
Structures of liminoids, divergokide R and divergokide S, tanshinones, and myricanol.

Limonoids have rich structural diversity and diverse biological activities, including anti-cancer, anti-atherosclerosis, anti-AIDS, antibacterial, and insecticidal properties [206,207,208,209] (Figure 14). To investigate the antimicrobial activity of limonin, Vikram et al. [210] isolated a series of limonoids (Figure 14) from grapefruit seeds and evaluated their potential to block cell-to-cell communication, biofilm formation, and T3SS expression in EHEC. Certain limonoid compounds have been confirmed to inhibit the intercellular communication, biofilm formation, and T3SS secretion of EHEC. The authors demonstrated that autoinducer-mediated signaling was inhibited by recording the loss of bioluminescence in *Vibrio harveyi* reporter strains, and the autoinducers included AHL and AI-2. All limonoids tested inhibited autoinducer-mediated intercellular signaling in a concentration-dependent manner. Obacunone was the most effective inhibitor of AHL, and AI-2 induced bioluminescence with IC_75_ values of 42.66 and 28.18 μM, respectively. Limonin and limonin glucoside IC_50_ values were compared, as IC_75_ values could not be obtained in the tested concentration range, for limonin and limonin glucoside were relatively less effective in inhibiting the autoinducer-mediated bioluminescence. All the limonoids demonstrated typical sigmoid activity against AHL- and AI-2-induced bioluminescence. The IC_50_ values for limonin and limonin glucoside against AHL activity were 138.04 and 223.87 μM, respectively. Limonin showed poor antagonistic activity against AI-2, with a distinct plateau at low concentrations. The IC_50_ values for limonin and limonin glucoside were 11.48 and 61.66 μM, respectively. Collectively, these results suggest significant inhibitory activity by obacunone and nomilin against cell–cell communication in the *V. harveyi* model [210].

The effects of isolated limonoids on biofilm formation were further investigated in both *V. harveyi* and EHEC, and it was found that these compounds inhibited biofilm formation in a dose-dependent manner in both Harveybacteria and EHEC [210].

The inhibitory activities of various limonoids were variable against *V. harveyi* as well as EHEC biofilm formation. Obacunone was the most effective antagonist of *V. harveyi* biofilm formation, with an IC_50_ of 91.2 μM. In addition, all the limonoids inhibited the formation of EHEC biofilm in a concentration-dependent manner. Obacunone was the most effective antagonist of EHEC O157:H7 biofilm formation, with 9–68% inhibition (IC_50_ ≈ 116.68 μM), and limonin and limonin glucoside inhibited EHEC biofilm formation by 7.15–33.64% and 0–19.55%, respectively, in the tested concentration range [210].

This study concluded that obacunone was the most potent antagonist of T3SSs, shiga toxin, and flagellar transcription in EHEC O157: H7 among the tested limonoids. Exposure to obacunone decreased the transcription of genes encoded in the LEE region by 2.23–5.16 fold, the transcription of *stx2* by 3.37-fold, and the transcription of flagellar genes by 2–3 fold. Obacunone did not affect the expression of *ptsN*. Limonin had moderate to no effect on the transcription of the genes studied; however, limonin induced the expression of *ptsN* moderately [210]. The results showed that some estadine properties could be helpful in alleviating EHEC infections and that they could be used as lead compounds in the development of new anti-infection drugs.

### 6.17. Divergokide R and Divergokide S

Ansamycins are a large group of macrocyclic lactam compounds, which are often derived from microbial fermentation, among which the anti-tumor geldanamycins, maytansinoids, and ansamitocins and the anti-tuberculosis rifamycins are famous examples [211,212,213] (Figure 14). Zhao et al. [214] isolated and elucidated five new divergolide congeners from *Streptomyces* sp. HKI0576. Among them, divergolides R and S significantly inhibited the secretion of the SPI-1 effectors SipA/B/C/D but did not affect FliC at a concentration of 100 μM [214]. At a concentration of 50 μM, divergolides R showed a concentration-dependent inhibitory effect, whereas divergolides S did not. Due to the weak cytotoxicity, the mechanism of action of divergolides R and S against T3SSs may be worthy of investigation [214].

### 6.18. Tanshinones

Tanshinones are derived from *Salvia miltiorrhiza*, which is widely used in the treatment of cardiovascular and cerebrovascular diseases. In 2019, Feng et al. [215] found that tanshinones (Figure 14) were effective inhibitors of the biogenesis of the T3SS needle of multidrug-resistant *P. aeruginosa*, based on a fluorescence-polarization-based assay designed by their group. Their tests confirmed that tanshinones are effectively competitive inhibitors of PscF, binding to PscE–PscG, with the following results: tanshinone 1 (TSN 1) IC_50_ = 2.15 μM, dihydrotanshinone 1 (dHTSN1) (IC_50_ = 0.68 μΜ), and dihydrotanshinone (dHTSN) (IC_50_ = 1.50 μM) [215]. Treatment of PAO1-infected mouse macrophages J774A.1 with three tanshinones at 100 μM showed neither inhibition of bacterial growth nor cytotoxicity to host cells [215]. In line with the biochemical assays, dHTSN and dHTSN1 were more active than TSN1 in terms of inhibitory activity against ExoS secretion. TSN1, dHTSN, and dHTSN1 inhibited *P. aeruginosa*-induced cell lysis dose-dependently but did not activate crpTSN in a concentration range of 0 to 100 μM. The survival of infected macrophages may be due to reduced activation of caspase-1, suggesting that tanshinones can inhibit T3SS. The lung burden of PAO1-infected mice treated with tanshinones was significantly improved. These results support the development of tanshinones as anti-infective drugs with anti-virulence effects.

### 6.19. Myricanol

Waxberry is a popular fruit from Southern China. In folk medicine, it is considered to lower blood pressure and have antibacterial properties, among others [216]. Myricanol (Figure 14) isolated from *Myrica nagi* is a natural product of cyclic diarylheptanoid and has antibacterial activity. Recently, according to the bioassay-oriented screening model, myricanol inhibited the secretion of the T3SS effector protein of *S. enterica* Serovar Typhimurium UK-1 χ8956 and inhibited infection of *S. typhimurium* in SW480 cells but did not affect the growth of bacteria and host cells [216]. The results showed that myricanol inhibited the secretion of SipC in a dose-dependent manner between 0 and 200 μM, with an IC_50_ value of 41.34 μM. By studying the inhibition mechanism, it was found that myricanol exhibited significant inhibitory activity against the disease-related SPI-1 gene, and a further study found that myricanol and HilD physically bound to block the DNA binding of HilD to the *hil*A and *inv*F genes [216]. This is a typical example of the dual use of medicine and food and the reason traditional Chinese medicine has protected the Chinese people for two thousand years.

### 6.20. Tannic Acid

**Figure 15:**
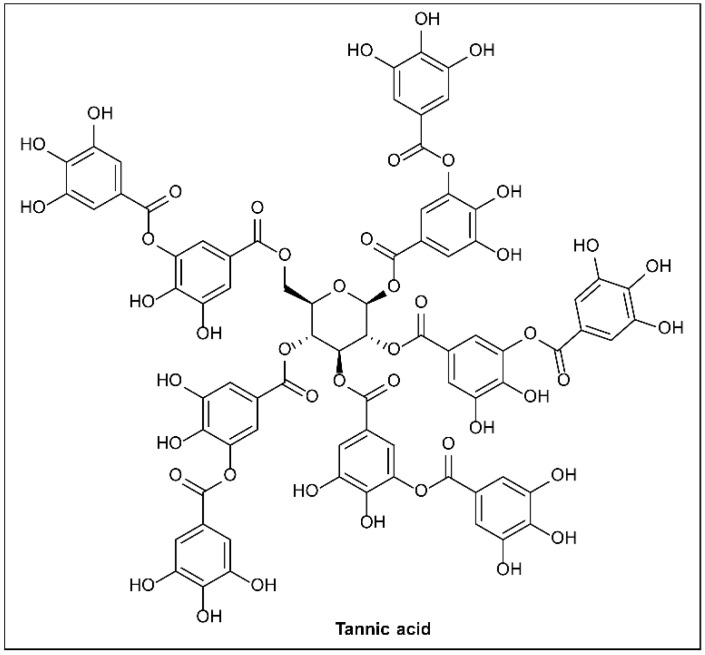
Structures of tannic acid.

Tannic acid (TA; Figure 15) is a kind of natural polyphenolic compound, which is derived from oak tree galls. It has anti-inflammatory, anti-viral, anti-fungal, and antioxidant activities and induces apoptosis in cancer cells, in addition to other biological activities [217]. Shu et al. [218] conducted a series of experiments to study TA’s T3SS inhibitory activity. Animal experiments showed that TA could significantly improve the survival rate of mice infected with *S. typhi*. Untreated mice died on the third day of infection, while seven days after infection TA-treated mice had a 40% higher survival rate than untreated mice. Anatomical analysis showed that the ceca of the TA-treated mice had only slight lesions, while the ceca of untreated mice showed obvious dehydration and atrophy [218]. The cytotoxicity and protective effects of TA on HeLa were verified by cell experiments. TA concentrations less than 16 μg/mL were not toxic to cells. Immunofluorescence experiments showed bacteria gathered around TA-untreated cells, whereas the number of bacteria around TA-treated cells was significantly reduced [218]. Furthermore, the experiment showed that TA had no significant effect on the growth of *S. typhimurium* at concentrations less than 32 μg /mL. Further studies showed that TA prevented bacterial infection by inhibiting the expression of SpiA-TEM. Notably, this inhibition is dose-dependent. To explore the mechanism of TA inhibiting SpiA expression, WT analysis found that TA dose-dependently inhibited structural component genes and effector genes of T3SS, including *hilC, hilD, hilA, rstA, invG*, *prgH, prgK*, and *prgI* [218]. In conclusion, TA can block bacterial infection and strongly inhibit T3SSs without affecting bacterial growth; therefore, it is a potential drug that can be used against drug-resistant bacteria.

## 7. Conclusions

Several small molecules, including synthesized and naturally occurring chemicals, have been found to exhibit T3SS inhibitory activities in recent years. Furthermore, researchers have made remarkable progress in understanding the functions, structures, and responses of host cells to the T3SS. However, because the T3SS is present in many Gram-negative pathogens and is itself a large protein complex, we do not know enough about it. Inhibitory mechanisms against small-molecule inhibitors are insufficient, and an inhibitor that works on the T3SS of one pathogen is likely to fail in another pathogen. In addition, because the T3SS regulation process is complex, it is not clear which part of the system small-molecule inhibitors act on, and this issue needs further study. Existing small-molecule inhibitors have not been particularly successful in inhibiting this system, and the inhibitory activity of these compounds is often the result of in vitro testing and rarely in vivo testing. Except for some synthetic compounds, to date, fluorothiazinon (FT) is the only small-molecule inhibitor of the T3SS that has entered the clinical stage; therefore, it is the one most likely to become a commercial small-molecule inhibitor of the T3SS. T3SS inhibitors of natural origin are usually scarce, making it difficult to further investigate their activity. In the future, research on the inhibition mechanism is required and a search for inhibitors with better activities that can overcome the existing problems in this field of study will allow more T3SS inhibitors to be considered as potential clinical drug candidates.

## Data Availability

Not applicable.
